# Electronic Structure and Surface Chemistry of Hexagonal Boron Nitride on HOPG and Nickel Substrates

**DOI:** 10.1021/acsomega.3c00562

**Published:** 2023-07-05

**Authors:** Didrik René Småbråten, Inger-Emma Nylund, Kenneth Marshall, Julian Walker, Maria Benelmekki, Mari-Ann Einarsrud, Joseph Kioseoglou, Sverre M. Selbach

**Affiliations:** † Department of Materials Science and Engineering, NTNU Norwegian University of Science and Technology, NO-7491 Trondheim, Norway; ‡ Department of Physics, Aristotle University of Thessaloniki, GR-54124 Thessaloniki, Greece

## Abstract

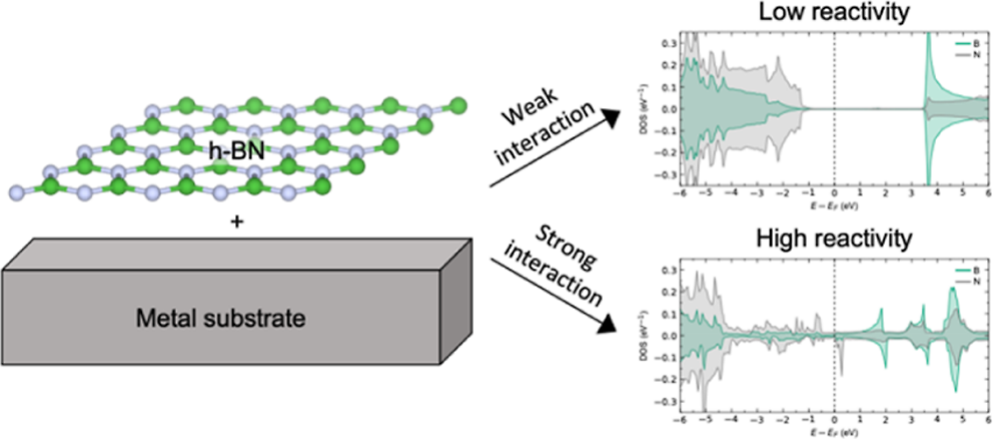

The effect of point defects and interactions with the substrate are shown by density functional theory calculations to be of significant importance for the structure and functional properties of hexagonal boron nitride (h-BN) films on highly ordered pyrolytic graphite (HOPG) and Ni(111) substrates. The structure, surface chemistry, and electronic properties are calculated for h-BN systems with selected intrinsic, oxygen, and carbon defects and with graphene hybrid structures. The electronic structure of a pristine monolayer of h-BN is dependent on the type of substrate, as h-BN is decoupled electronically from the HOPG surface and acts as bulk-like h-BN, whereas on a Ni(111) substrate, metallic-like behavior is predicted. These different film/substrate systems therefore show different reactivities and defect chemistries. The formation energies for substitutional defects are significantly lower than for intrinsic defects regardless of the substrate, and vacancies formed during film deposition are expected to be filled by either ambient oxygen or carbon from impurities. Significantly lower formation energies for intrinsic and oxygen and carbon substitutional defects were predicted for h-BN on Ni(111). In-plane h-BCN hybrid structures were predicted to be terminated by N–C bonding. Substitutional carbon on the boron site imposes n-type semiconductivity in h-BN, and the n-type character increases significantly for h-BN on HOPG. The h-BN film surface becomes electronically decoupled from the substrate when exceeding monolayer thickness, showing that the surface electronic properties and point defect chemistry for multilayer h-BN films should be comparable to those of a freestanding h-BN layer.

## 1 Introduction

The interest in 2D materials, such as graphene and transition metal dichalcogenides, has increased tremendously the last decades.^1−3^ This family of 2D materials has excellent properties, which can be utilized in new device concepts.^1,4−9^ Among these 2D materials, hexagonal boron nitride (h-BN) is the only insulator (*E*_g_ ∼ 6 eV^10,11^) and has a crystal structure analogous to that of graphene. h-BN also offers high transparency, high thermal conductivity, and a broad range of absorption wavelengths.^11^ These properties can be optimized by doping or functionalization^12−17^ for applications in, *e.g.*, electronics, optoelectronics, and sensors. Furthermore, these 2D materials can be grown as thin films uniformly covering large areas, facilitating their integration into electronic circuitry.

Deposition methods of h-BN thin films are well established, including physical vapor deposition techniques such as molecular beam epitaxy,^18,19^ pulsed laser deposition,^20−22^ and chemical vapor deposition.^23−26^ Several substrates have been demonstrated to be suitable for deposition of h-BN, such as highly ordered pyrolytic graphite (HOPG),^20^ silicon wafers,^21^ sapphire,^20^ Ag(111)/SrTiO_3_(001),^21^ and Cu foil. Graphene has been the most used substrate for h-BN growth due to the relatively small lattice mismatch of 1.8%.^27,28^ h-BN/graphene heterostructures - in-plane, out-of-plane, and in nanoribbons- have also been extensively studied,^29−37^ mostly for applications within electronic devices due to their similar crystal structures and distinctly different electronic properties.^1,11^ Metallic substrates have great advantages of low cost, availability, scalability, and compatibility with roll-to-roll processing.^38^

Vacancy engineering in h-BN is established as an efficient strategy to increase the catalytic performance. The band gap of h-BN can be significantly reduced by introducing impurities or B- and N-vacancies, enhancing the electrocatalytic activity. In addition, theoretical and experimental investigations have shown that the thermodynamically favorable formation of B-vacancies acts as active sites for oxygen adsorption.^39^ It is theoretically established that the substitution of oxygen at the nitrogen position in h-BN reduces the band gap from 4.56 to about 4.34 eV depending on the concentration of substituted oxygen.^40^ Furthermore, a specific complex defect containing nitrogen in boron position near a nitrogen vacancy sites is found to establish quantum emission in h-BN.^41,42^ Moreover, experimentally, it is also found that O atoms doped in h-BN nanosheets form B–O bonds.^43^

Recently, several experimental and theoretical studies are focused on the interaction between doped or undoped h-BN and molecular oxygen. A defect-free “inert” h-BN surface functionalized by weakly interacting metallic atoms, such as Au and Au_2_, experiences significant changes to the binding and catalytic activation of the O_2_ molecule.^44^ Moreover, N impurities make h-BN highly active for O_2_ adsorption due to the introduction of defect states near the Fermi level,^45^ and N-terminated defects are also less stable than O-terminated defects.^46^ An oxygen atom migrating on the h-BN surface prefers to stay on top of an N atom, with its migration path restricted by three adjacent B atoms. In addition, the B–N bond is found to be stretched and under certain conditions may even break due to chemisorption of an oxygen atom.^47^ Finally, concerning oxygen doping, it is concluded that O atoms can repair the nitrogen-defected lattice sites, while multiple oxygen impurities will lead to h-BN lattice distortion.^48^

In the case of carbon doping, it is found that carbon atoms lead to reduction of the h-BN band gap and cause an insulator to semiconductor transition.^49,50^ First-principles calculations concluded that carbon atoms preferentially substitute boron atoms in the h-BN lattice and that this substitution is favored by electron removal. The boron substitution by carbon corresponds to single electron doping of h-BN, which leads to n-type semiconductivity,^51−53^ as well as considerable catalytic activity in a large area that extends far away from the carbon impurity. Moreover, the adsorption energy of molecular oxygen decreases sluggishly with the increase in distance from the carbon impurity in the boron position. Various atoms of group III, IV, and V and transition metal elements, such as B, N, Al, Si, Ge, Ni, Pt, Pd, and Au, are examined as dopants, and no similar effect is observed for monolayer h-BN. Consequently, Gao *et al.* concluded that even small concentrations of carbon atoms can activate a significant surface area of monolayer h-BN, converting it to a promising catalytic material.^54^ In addition, recently, visible single-photon emitters (SPEs) were identified in carbon-doped h-BN. Computational analysis of the simplest carbon-related complex defects established the negatively charged defect in which C substitutes N and a vacancy in the neighboring B site defects as SPEs.^55^ However, Alcántara Ortigoza and Stolbov examined seven possible carbon-related defects and concluded that the defects where C substitutes B (C_B_) and C substitutes N (C_N_) have the lowest formation energies and that the C_N_ defect has an excitation spectrum in agreement with the observed SPE.^56^

Numerous studies on atomic-scale functional defect modulations in several 2D materials^42,50,57−61^ have been reported. Density functional theory (DFT) calculations have provided a fundamental understanding of the structural configurations and electronic properties of h-BN on a wide range of metal substrates (see, *e.g.*, the review in ref (62) and references therein). Particular attention has been given to the interaction between h-BN and Ni(111) surfaces,^17,63−71^ where monolayers of h-BN are typically found to be chemisorbed on the surface. The charge transfer between the film and the substrate closes the band gap and renders monolayer h-BN metallic.^65^ This alters the reactivity of an otherwise chemically inert h-BN surface, which is important for the catalytic behavior.^67−71^

The current theoretical studies on metal substrate-supported h-BN focus mainly on the surface chemistry for pristine h-BN layers with or without adsorbed ad-atoms. Point defects in h-BN, on the other hand, are investigated mainly in freestanding monolayers or in bulk h-BN. However, since h-BN films will contain point defects after deposition, a fundamental understanding of substrate–film–defect interactions is crucial for understanding and controlling the functional properties of metal-supported layered h-BN thin films. Here, we study the effect of intrinsic and substitutional defects on the crystal structure and electronic properties of h-BN on HOPG and Ni(111) metal substrates by DFT calculations. h-BN is predicted to be HOPG tolerant, while Ni(111) suppresses the electronic band gap rendering monolayer h-BN conducting. The differences in electronic properties affect the defect formation energies, where h-BN Ni(111) shows significantly lower defect energies than freestanding h-BN and h-BN on HOPG.

## 2 Methods

**Figure 1:**
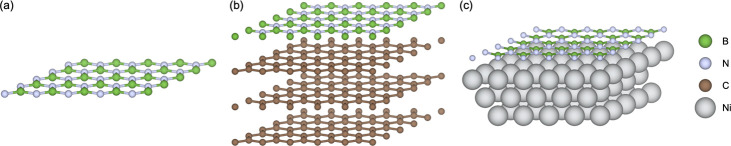
DFT model systems used in the majority of the study for addressing (a) freestanding h-BN monolayer, (b) h-BN on HOPG, and (c) h-BN on Ni(111), illustrated in the *p*(5 × 5) supercell size. The entire 20 Å vacuum region is not shown, for clarity.

DFT calculations were performed with the projector augmented wave (PAW) method^72^ as implemented in VASP.^73,74^ The van der Waals (vdW) functional^75−77^ rev-vdW-DF2^78^ was used to describe the weak vdW bonding. B (2s, 2p), N (2s, 2p), C (2s, 2p), Ni (3p, 3d, 4s), and O (2s, 2p) were treated as valence electrons, with a plane-wave energy cutoff of 500 eV. The electron occupancy was described using the tetrahedron method with Blöchl corrections for bulk h-BN, a second-order Methfessel–Paxton^79^ smearing of 0.2 eV for bulk nickel and graphite, and a Gaussian smearing of 0.05 eV for the heterostructures. The B, N, C, and O atoms were initialized with zero magnetic moments, while Ni atoms were initialized with 2 μ_B_ and ferromagnetic order. The Brillouin zone was sampled with a Γ-centered *k*-point grid with a density of 15 × 15 × 5 for bulk h-BN and graphite, 11 × 11 × 11 for bulk nickel, 15 × 15 × 1 for the *p*(1 × 1) heterostructures, and 3 × 3 × 1 for the *p*(5 × 5) heterostructures. The *k*-point sampling was doubled for the density of states (DOSs) calculations. A force criterion for structural relaxation of 0.001 eV Å^–1^ was used for the pristine h-BN supercells and 0.01 eV Å^–1^ for the defect cells. Dipole corrections were included when relaxing the asymmetric slabs. The structural configurations and electronic properties of the pristine h-BN/metal heterostructures were modeled by *p*(1 × 1) supercells of h-BN on top of three atomic layer thick metal slabs separated by a vacuum spacing of 20 Å. The freestanding monolayer was modeled using the relaxed bulk h-BN lattice parameter, while the heterostructures were modeled using the average h-BN and respective substrate lattice parameters, as described in ref (80). The lowest energy heterostructure configurations are used (see Section 3.1 and Supporting Note 3), as illustrated in Figure 1. The defect chemistry was modeled by the corresponding *p*(5 × 5) supercells. The bottom two atomic layers were fixed, and the top layer and the film monolayer(s) (ML) were relaxed. A comparison between the reported *p*(1 × 1) h-BN/metal heterostructure results and those obtained with seven atomic layer thick metal slabs with a vacuum spacing of 40 Å using the hard B, N, and C PAW potentials is given in Supporting Note 4. Binding energies for bulk h-BN and graphite were calculated by the energy difference between the relaxed bulk structures and two ML separated by a 20 Å vacuum region, while the binding energies for adsorbed h-BN on the metal substrates were calculated from the energy difference between 1 ML of h-BN adsorbed on the surface and placing 1 ML of h-BN in the middle of the vacuum region. Defect formation energy assuming charge neutral cells are calculated by
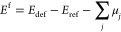
1where *E*_def_ is the calculated total energy for a defect supercell, *E*_ref_ is the total energy for a corresponding reference system with pristine h-BN, and μ_*j*_ is the chemical potential of species *j*. In the following, μ_B_ is defined as the total energy per atom in bulk α-B, while μ_N_ is defined by the chemical potential of N_2_(g), . At thermodynamic equilibrium, the formation energy of h-BN is defined by Δ*H*^f^(h-BN). Here, we consider N-rich conditions since N_2_(g) could be present during the film deposition if the vacuum condition is imperfect.^81^ μ_O_ is defined by , and μ_C_ is defined by the total energy for bulk graphite. Chemical potentials for the gaseous species are taken from thermochemical data at 298 K.^82^ The DOSs were analyzed with sumo^84^ and plotted with a Gaussian broadening of 0.025 eV for increased readability. All crystal structure illustrations were prepared with VESTA.^83^

## 3 Results and Discussion

### 3.1 Assessment of the Van der Waals Functionals

**Figure 2:**
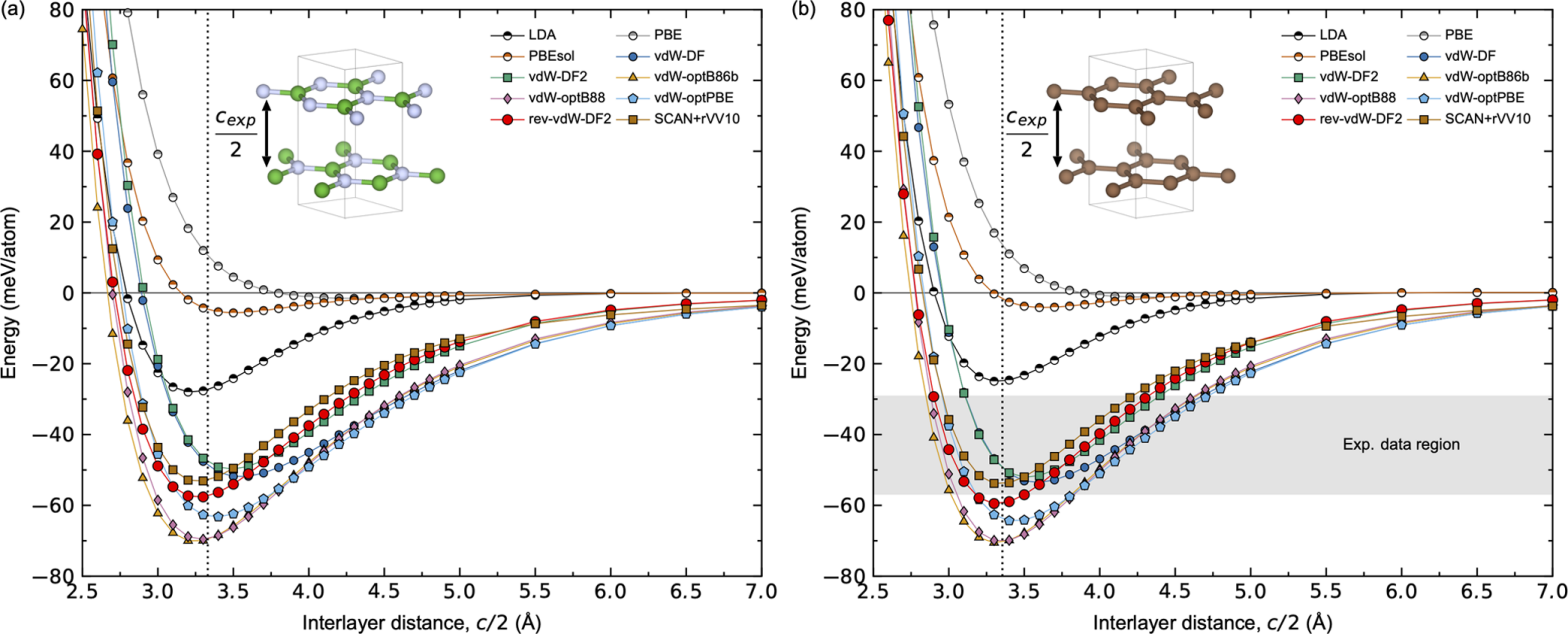
Calculated binding energy as a function of interlayer distance *c*/2 for (a) h-BN (*P*6̅*m*_2_) and (b) graphite (*P*6_3_/*mmc*) for the different functionals investigated. The *a* lattice parameter was fixed to the experimental bulk parameters of h-BN and graphite for all calculations. The experimentally reported binding energies of graphite are 31 ± 2, 43, 52 ± 5, and 35 (+15 to −10) meV per atom, as described in ref (91). The results using the rev-vdW-DF2 functional are marked by red circles. The dotted vertical lines correspond to the experimental interlayer distances *c*_exp_/2.

The assessment of the vdW functional was done by DFT calculations using VASP,^72−74^ with seven different vdW functionals,^75,76,78,85−87^ the PBEsol functional,^88^ and the standard PBE^89^ and LDA^90^ functionals (see the legend in Figure 2). We first evaluate the vdW functionals by comparing calculated and experimentally reported lattice parameters and binding energies of h-BN in the two stable polymorphs (*P*6̅*m*_2_ and *P*6_3_/*mmc*) and of graphite (*P*6_3_/*mmc*). The calculated lattice parameters and interlayer binding energies are summarized in Tables 1 and S1–S3. All functionals investigated give in-plane lattice parameters within 1% deviation from the experimental lattice parameters. The *c* lattice parameter is however very sensitive to the choice of the functional, where the rev-vdW-DF2 and vdW-optPBE functionals give reasonable *c* lattice parameters for the two h-BN polymorphs and for graphite. To further assess the vdW functionals, we have calculated the interlayer binding energy, *e.g.*, the attractive energy between the atomic layers, as a function of interlayer distance for h-BN and graphite in Tables S1–S3 and Figure 2, with fixed experimental *a* lattice parameter. The rev-vdW-DF2 functional gives the best binding energy of graphite, a good description of the *c* lattice parameter of graphite and h-BN, and reproduces the bulk Ni lattice parameter within 1% from the experimental value (Table S4). The remaining results are obtained using the rev-vdW-DF2 functional.

**Table 1:** Calculated Binding Energies, *E*_b_, Binding Height, *h*, and Electronic Band Gap, *E*_g_, for Bulk h-BN, 1 ML of h-BN on HOPG, and 1 ML of h-BN on Ni(111)

system	*E*_b_ (meV/h-BN)	*h* (Å)	*E*_g_ (eV)
bulk h-BN	–57.62^a^	3.24^a^	4.36
HOPG	–60.21	3.27	4.28
Ni(111)	–101.41	2.11	metallic

a Calculated for bulk h-BN, see Tables S1 and S4.

Next, we determine the most stable configuration of h-BN on the HOPG and Ni(111) surfaces (see Supporting Note 2). We identify six most probable configurations on the two substrates, shown in Figures S1 and S2, respectively. After structural relaxation, we identify one clear energetically favored h-BN/Ni(111) configuration, where B sits on the hollow Ni_fcc_ site and N on the Ni_top_ site as previously reported.^17,63−71^ For h-BN on HOPG, we find two configurations with similar binding energies that are comparable to previous work on graphene on h-BN.^33^ The configuration that most closely mimics the lowest energy bulk “A–B” stacking of h-BN and graphite was chosen as a model system in the following.

### 3.2 Pristine h-BN

**Figure 3:**
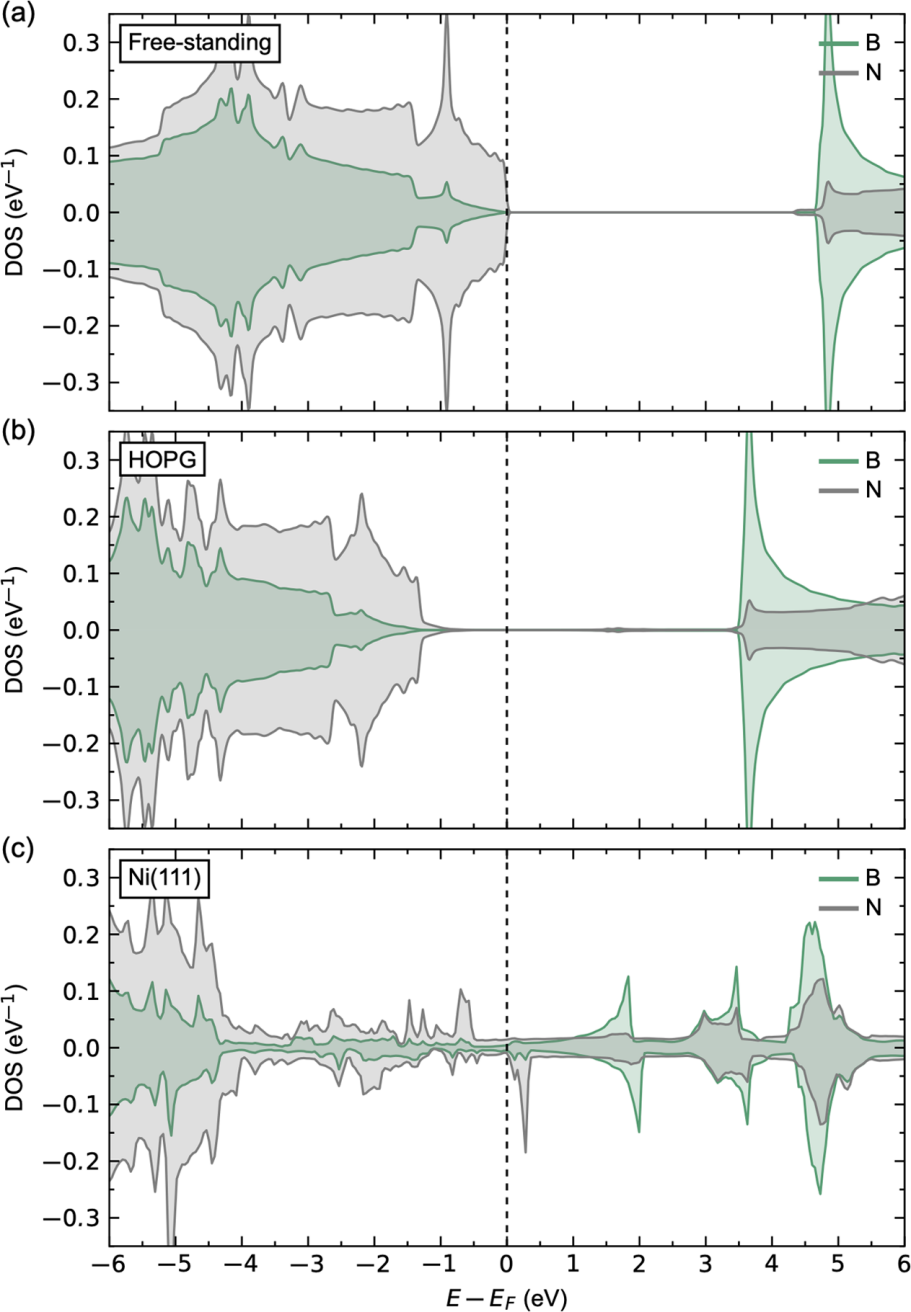
Calculated local electronic structure (LDOS) for (a) freestanding monolayer of pristine h-BN, (b) 1 ML of pristine h-BN on HOPG, and (c) 1 ML of pristine h-BN on Ni(111).

First, we determine the coupling between pristine h-BN and the substrate. A comparison of the local DOS (LDOS) for a monolayer (1 ML) of freestanding h-BN, 1 ML of h-BN on HOPG, and 1 ML of h-BN on Ni(111) is shown in Figure 3. The LDOS for a freestanding h-BN with a calculated band gap of 4.36 eV is shown in Figure 3a. The resulting band gap is lower than the experimental one (*E*_g_ ∼ 6 eV^10,11^), as expected since DFT is known to underestimate band gaps.

The calculated LDOS for 1 ML of h-BN on HOPG is shown in Figure 3b. The h-BN layer is predicted to be insulating from the LDOS, similar to that of the freestanding h-BN, with a band gap of 4.28 eV. This is as expected since there are only weak vdW forces acting between the h-BN layer and the HOPG substrate and thus negligible out-of-plane orbital interaction between them. The weak vdW bonding is also apparent from the resulting binding height of 3.27 Å, comparable to the binding height of bulk h-BN (Table 1). Furthermore, we find a binding energy of −60.21 meV/h-BN, which is also comparable to the bulk h-BN value. This indicates physisorption of h-BN on HOPG. Adding multiple monolayers on top of the 1 ML h-BN/HOPG surface does not alter the electronic properties (Figure S4), nor the binding heights (Table S7). h-BN is thus expected to be completely decoupled electronically from the HOPG surface and should act as bulk-like h-BN regardless of the film thickness. Some unoccupied states within the band gap about 1 eV above the Fermi level are also observed. These relative differences are, however, subtle in the order of 0.005 eV/h-BN, which suggest a weak out-of-plane orbital interaction between h-BN and the substrate.

Figure 3c shows the calculated LDOS for 1 ML of h-BN on top of Ni(111), leading to metallic-like behavior of the h-BN layer, contrary to the wide band gap semiconducting behavior for 1 ML of h-BN on HOPG, as previously reported both experimentally and theoretically.^65^ A significant overlap between the N 2p_*z*_ and Ni 3d_*z*^2^_ and 3d_*xy*_ orbitals around the Fermi level is observed, as shown in the orbital resolved LDOS in Figure S7b,c. This orbital overlap also induces a weak magnetism in h-BN, where N shows a small induced magnetic moment of 0.019 μ_B_, accompanied by a reduction in the magnetic moment of Ni from 0.704 μ_B_ at the top Ni layer for the clean Ni(111) surface to 0.619 μ_B_ at the top Ni layer in the h-BN/Ni(111) heterostructure. The coupling between B and Ni is on the other hand weak, where no significant orbital overlap can be inferred from the orbital-resolved LDOS (Figure S7a). The binding height is 2.11 Å (Table 1), which indicates chemisorption rather than physisorption of h-BN on the Ni(111) surface. This is further supported by a significantly more negative binding energy of −101.41 meV/h-BN. By adding multiple layers of h-BN on top of the 1 ML h-BN/Ni(111) heterostructure, all the added layers are structurally and electronically decoupled from the substrate, which becomes apparent from the calculated bulk-like binding heights of ∼3.3 Å (Table S8) and a wide band gap LDOS (Figure S4). The surface of a multilayer-thick h-BN film is thus predicted to be completely decoupled from the Ni(111) substrate, in agreement with a previous work.^65^

These results indicate that the electronic structure of a pristine monolayer of h-BN can be significantly different depending on the choice of the substrate. Hence, the different systems are expected to show different reactivities and defect chemistries, which will be addressed in the following. The surface of multilayer h-BN films, on the other hand, are found to be decoupled from the substrates.

### 3.3 Intrinsic Defect Chemistry

Next, we address the intrinsic defect chemistry of h-BN with respect to the substrate. Table 2 shows a summary of the formation energies for boron (v_B_) and nitrogen vacancies (v_N_). The formation energies for the freestanding monolayer are comparable to those reported in the literature,^92^ where the energy preference for v_B_ over v_N_ is reproduced. The same trend in energy preference for v_B_ over v_N_ is observed for h-BN on Ni(111), while the opposite trend is observed for h-BN on HOPG. The formation energies of vacancies in freestanding h-BN and h-BN on HOPG systems are comparable, while we find that vacancies in h-BN on Ni(111) cost much less energy. The reduction in the formation energies for h-BN on Ni(111) can be explained by the inherent charge transfer between the h-BN layer and the substrate described above, where the excess charges associated with the vacancies can readily be electronically screened, as further described below.

**Table 2:** Calculated Defect Formation Energies for Vacancies (), Oxygen Substitution (), and Carbon Substitution () at the Two Different Lattice Sites *j* under N-Rich Conditions^a^

site *j*	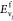 (eV)			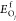 (eV)			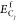 (eV)		
	h-BN	HOPG	Ni(111)	h-BN	HOPG	Ni(111)	h-BN	HOPG	Ni(111)
B	7.59	7.55	2.81	5.82	6.12	4.48	1.75	0.51	0.85
N	7.84	6.86	3.66	1.55	–0.68	–0.74	4.43	4.66	2.43
Δ*E*^f^	0.25	–0.70	0.85	–4.26	–6.80	–5.21	2.68	4.15	1.58

a The difference in formation energy between the sites, Δ*E*^f^ = *E*_N_^f^ – *E*_B_^f^, are also shown, where negative values indicate a preference for the N-site.

**Figure 4:**
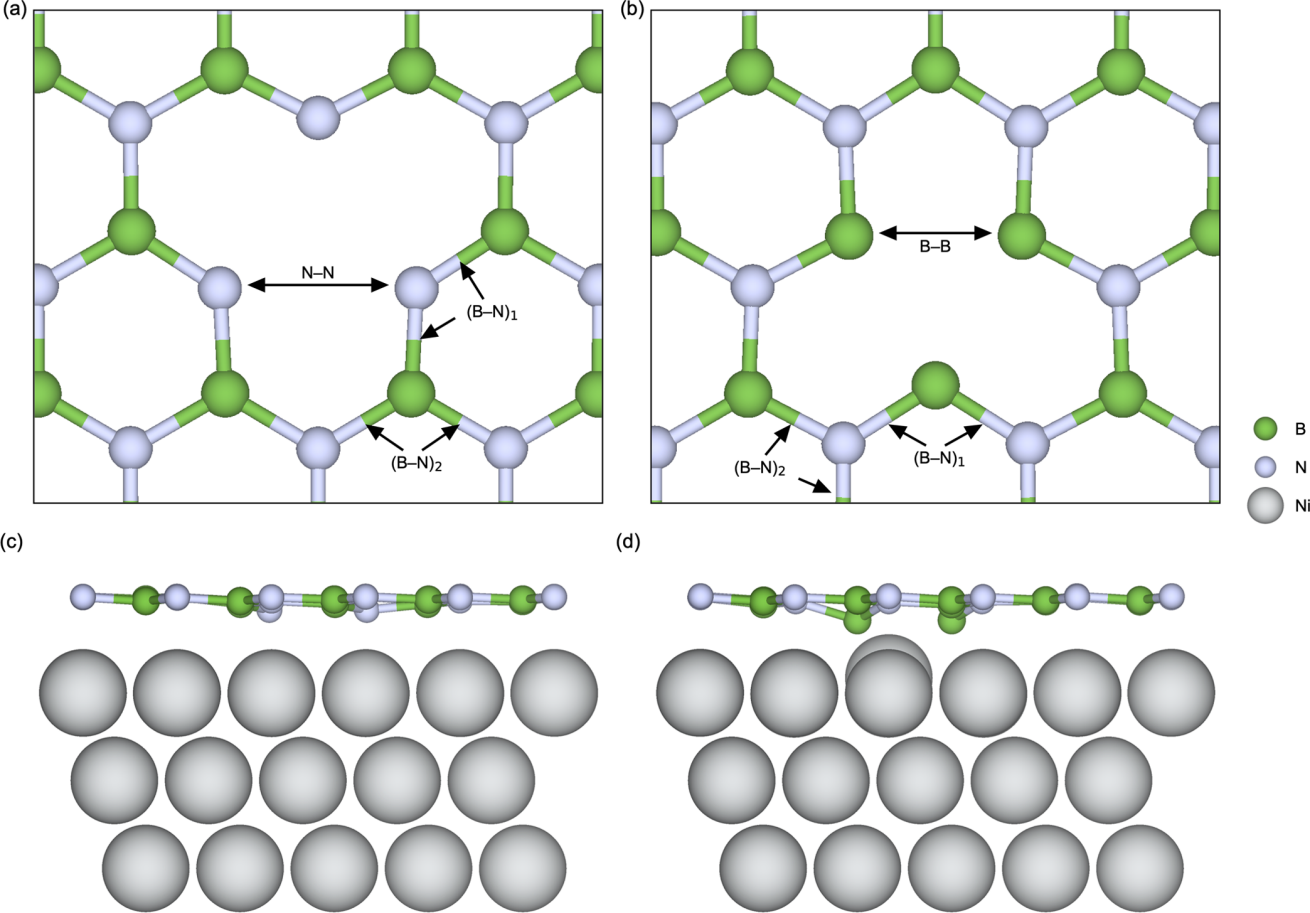
Top view of the local crystal structure in the vicinity of (a) v_B_ and (b) v_N_ in freestanding h-BN. Side view of (c) v_B_ and (d) v_N_ in 1 ML h-BN on Ni(111).

Structurally, v_B_ tends to induce a local in-plane expansion, while v_N_ tends to induce a local in-plane contraction. This expansion or contraction can be quantified by changes in the bond lengths in the vicinity of the defects, as illustrated for freestanding h-BN in Figure 4a,b (all v_B_ and v_N_ structures are visualized in Figures S9 and S10). The resulting bond lengths are summarized in Table 3. For v_B_ in freestanding h-BN and h-BN on HOPG, we find longer N–N bond lengths of, respectively, 2.64 and 2.58 Å surrounding v_B_, compared to the bulk values of 2.51 and 2.49 Å. In contrast, we find shorter B–B bond lengths of, respectively, 2.32 and 2.39 Å surrounding v_N_. The defect geometries in the freestanding monolayer are in qualitative agreement with a previous work.^93^ The structural perturbations for the intrinsic defects in h-BN on Ni(111) are more complex. Compared to the bulk B–B/N–N bond lengths of 2.49 Å, we find longer N–N bond lengths of 2.57 Å surrounding v_N_ and unaltered B–B bond lengths of 2.49 Å. However, we also find significant structural perturbations parallel to the h-BN layer, where the surrounding atoms are protruding out of the h-BN layer toward the Ni surface as illustrated in Figure 4c,d. This is especially prominent for the B-atoms surrounding v_N_ in Figure 4d, where we also find that the Ni-atom at the center of the vacancy is protruding out of the substrate. This results in significantly shorter B–Ni bond lengths of 1.95 Å compared to 2.53 Å for pristine h-BN on Ni(111), which in turn indicates stronger orbital interactions between Ni and B. v_B_ induces a small contraction of the B–N bonds in the second coordination shell relative to that in bulk in the order of 0.04–0.05 Å for all systems, while no significant perturbations in the third coordination shell is observed. For v_N_, both freestanding monolayer h-BN and h-BN on Ni(111) show a small expansion of the B–N bonds in the second coordination shell of 0.01–0.02 Å, while a small contraction of 0.02 Å is observed for h-BN on HOPG. No significant changes in the third coordination shells are observed. Note that intrinsic defects in h-BN are reported to break the three-fold symmetry due to the (pseudo) Jahn Teller effect.^93,94^ Such effects are not allowed in the present study since the defects are initialized with perfect symmetry. Since all defects here are symmetric, the general trends with respect to the choice of the substrate should still hold.

**Table 3:** Calculated Bond Lengths Surrounding v_B_ and v_N_^a^

bond	bond length (Å)					
	defect v_B_			defect v_N_		
	h-BN	HOPG	Ni(111)	h-BN	HOPG	Ni(111)
B–B/N–N	2.68	2.58	2.57	2.32	2.39	2.49
(B–N)_1_	1.41	1.40	1.39	1.46	1.42	1.47
(B–N)_2_	1.46	1.45	1.46	1.46	1.45	1.44
(B–B/N–N)_bulk_	2.51	2.49	2.49	2.51	2.49	2.49
(B–N)_bulk_	1.45	1.44	1.44	1.45	1.44	1.44

a Bulk B–B, N–N, and B–N bond lengths are shown for comparison.

The binding heights for v_B_ and v_N_ for the HOPG and Ni(111) systems are also summarized in Table 4, which are comparable to those for pristine h-BN. The binding heights for the protruding boron or nitrogen atoms for h-BN on Ni(111) in Figure 4c,d are marked in parenthesis.

**Table 4:** Calculated Binding Heights *h* for Vacancies (v_*j*_), Oxygen Substitution (O_*j*_), and Carbon Substitution (C_*j*_) at the Two Lattice Sites *j*^a^

site *j*	binding height *h* (Å)					
	v_*j*_		O_*j*_		C_*j*_	
	HOPG	Ni(111)	HOPG	Ni(111)	HOPG	Ni(111)
B	3.25	2.13 (1.90)	3.25	2.13 (1.88)	3.21	2.13 (2.27)
N	3.20	2.10 (1.65)	3.19	2.12 (1.88)	3.21	2.13 (2.07)
pristine h-BN	3.27	2.11	3.27	2.11	3.27	2.11

a The binding heights for the protruding boron and nitrogen atoms for h-BN on Ni(111) in Figures 4c,d, 6c,d, and 9c,d, respectively, are given in parenthesis. The reference binding heights for pristine h-BN are shown for comparison.

**Figure 5:**
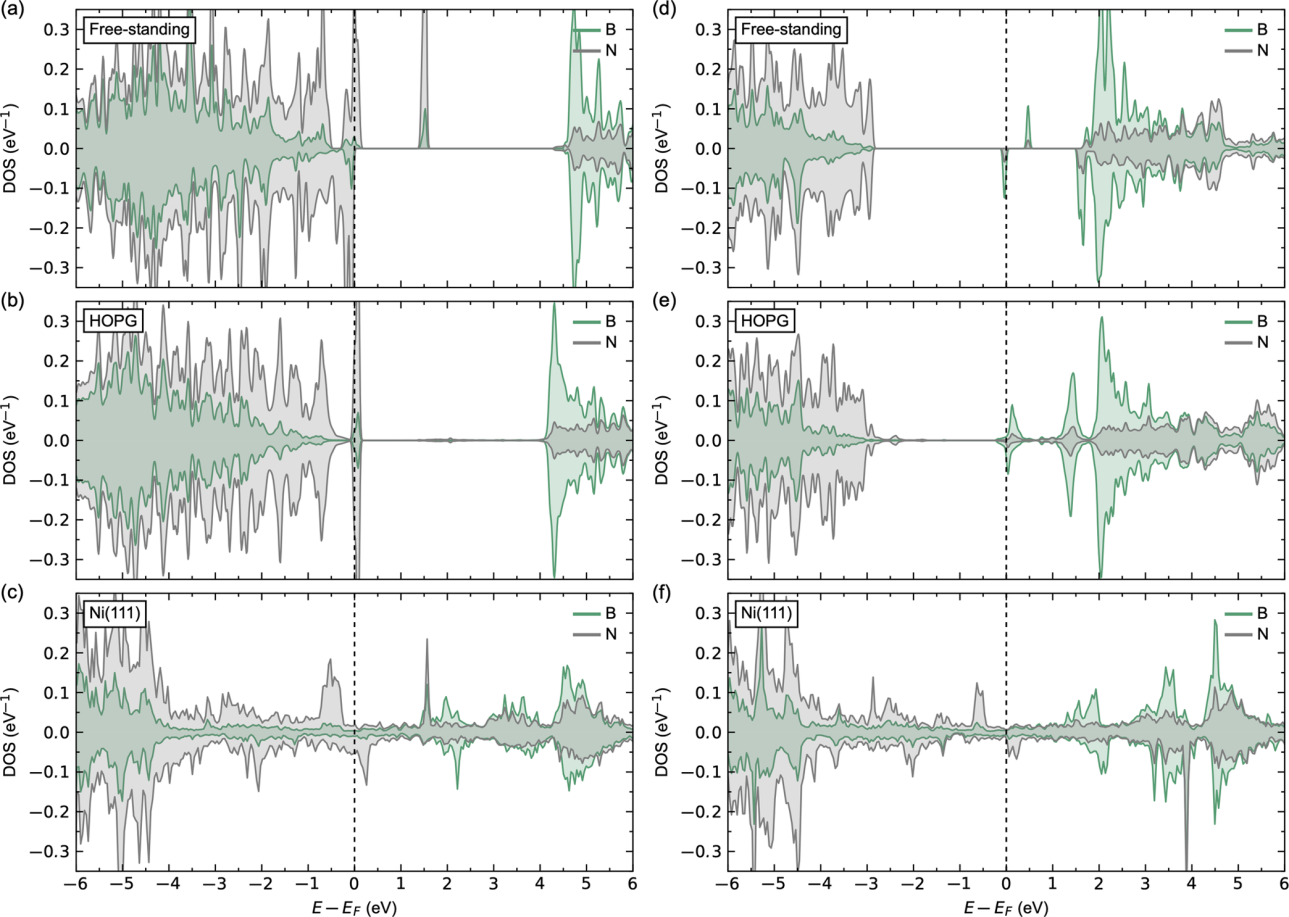
Calculated LDOS for (a–c) v_B_ and (d–f) v_N_ in (a,d) freestanding h-BN monolayer, (b,e) 1 ML of h-BN on HOPG, and (c,f) 1 ML of h-BN on Ni(111).

The LDOSs for h-BN with v_B_ or v_N_ are shown in Figure 5a–c and d–f, respectively. v_B_ is expected to behave like a triple acceptor defect. The calculated LDOSs for v_B_ in the freestanding h-BN sheet in Figure 5a reveal deep unoccupied acceptor states in the band gap of N 2p character, in line with previous reports.^93−95^ The defect induces local magnetism, where the nitrogen atoms surrounding v_B_ show magnetic moments 0.514–0.592 μ_B_. For v_B_ in h-BN on HOPG in Figure 5b, we find a shift in the Fermi level, however, with no unoccupied acceptor states in the band gap. No induced magnetization is observed. This can be reasoned from the weak film/substrate electronic coupling described above. Finally, for v_B_ in h-BN on Ni(111) in Figure 5c, we observe no qualitative changes compared to pristine h-BN on Ni(111). The donated holes localize on the surrounding nitrogen atoms and the surface nickel atoms below the defect. This becomes apparent from a small increase in the N magnetic moments from 0.019 μ_B_ in pristine h-BN on Ni(111) to 0.108–0.115 μ_B_ for v_B_ in h-BN and Ni(111) and a small increase in Ni magnetic moments below the defect from 0.619 to 0.640–0.660 μ_B_.

v_N_ is expected to behave like a triple-donor defect. The calculated LDOSs for v_N_ in the freestanding h-BN sheet in Figure 5d reveal occupied states in the band gap with mainly B 2p character as reported in the literature.^93,94,96^ The localization of the donated electrons on the surrounding boron atoms is further supported by the induced B magnetic moments of 0.093–0.094 μ_B_. A similar LDOS is also found for v_N_ in h-BN on HOPG in Figure 5e, with occupied donor state in the band gap below the conduction band minimum (CBM). As for v_B_ in h-BN on HOPG, no change in magnetization is observed. No qualitative changes in LDOS for v_N_ in h-BN on Ni(111) (Figure 5f) compared to pristine h-BN on Ni(111) is observed. The donated electrons are localized mainly on the nickel situated beneath the v_N_ site, apparent from a significant decrease in the Ni magnetic moment from 0.619 μ_B_ in the pristine cell to −0.010 μ_B_ in the defect cell.

The changes in total magnetization with v_B_ relative to pristine h-BN are 2.221 and 0.553 μ_B_ for the freestanding monolayer and h-BN/Ni(111), respectively, while no change is observed for v_B_ in h-BN/HOPG as described above. The corresponding changes in total magnetization with v_N_ are 0.410, 0.043, and −1.336 μ_B_ for the freestanding monolayer, h-BN/HOPG, and h-BN/Ni(111), respectively. The relatively higher total magnetization for v_B_ compared to v_N_ in the freestanding monolayer is comparable to that reported for similar defect–defect distances in the literature.^94^ However, their magnitudes are different from the values reported in ref (94). A detailed description of defect-induced magnetization requires further in-depth theoretical studies beyond the scope of this work.

### 3.4 Oxygen Defect Chemistry

Next, we address the oxygen defect chemistry with respect to the choice of the substrate. Several studies report significant h-BN_*x*_O_*y*_ bonding in deposited films, which is typically attributed to substitution of nitrogen by oxygen atoms.^97^ To corroborate this, we investigate the substitution of boron and nitrogen with oxygen (O_B_ and O_N_), and the defect formation energies for the different systems are summarized in Table 2. We find formation energies for O_B_ of 6.48, 6.78, and 5.13 eV in freestanding h-BN, h-BN on HOPG, and h-BN on Ni(111), respectively. The corresponding formation energies for O_N_ of +1.58, −0.66, and −0.72 are ∼5–7 eV lower in energy than O_B_. This can be reasoned from the large structural perturbations associated with substituting a cation boron site with an oxygen anion, as described further below. The formation energies for O_B_ and O_N_ are lower than those for v_B_ and v_N_ (Table 2), which indicates an energetic driving force for filling any inherent vacancies present in the film after deposition with oxygen present in the deposition chamber or upon exposure to air.

**Figure 6:**
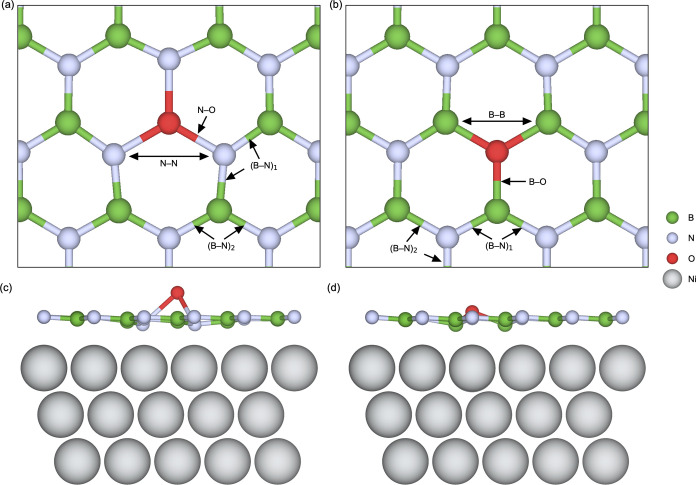
Top view of the local crystal structure in the vicinity of (a) O_B_ and (b) O_N_ in freestanding h-BN. Side view of (c) O_B_ and (d) O_N_ in 1 ML h-BN on Ni(111).

The local crystal structures for O_B_ and O_N_ are illustrated for the freestanding monolayer in Figure 6a,b, respectively (all O_B_ and O_N_ structures are visualized in Figures S11 and S12). Locally, O_B_ and O_N_ both induce local structural expansion, as apparent from the bond lengths surrounding the defects summarized in Table 5. The resulting N–O and B–O bond lengths in the vicinity of, respectively, O_B_ and O_N_ are greater than the bulk bond lengths. Additionally, both defects give increased N–N and B–B distances in the vicinity of the defects. These structural perturbations are most pronounced for O_B_, which indicates a smaller local stress for O_N_, in agreement with the defect formation energies described above. Both defects give a small local contraction of the B–N bonds in the first coordination shell relative to the bulk of −0.03 Å for both the freestanding monolayer and for h-BN on HOPG. For 1 ML h-BN/Ni(111), on the other hand, oxygen is protruding out of the surface as visualized in Figure 6c,d, accompanied by the nearest nitrogen or boron atoms moving toward the surface. This results in a net contraction of the B–N bonds in the first coordination shell of −0.03 Å for O_B_ and a net expansion of +0.02 Å for O_N_. The protruding oxygen is more pronounced for O_B_, which further elucidates the lower stability of O_B_ compared to O_N_ in h-BN on Ni(111) described above. No significant lattice perturbations in the second coordination shells are observed. The binding heights for O_B_ and O_N_ for the HOPG and Ni(111) systems are summarized in Table 4, which are comparable to those for pristine h-BN. The binding heights for the protruding boron or nitrogen atoms for h-BN on Ni(111) in Figure 6c,d are marked in parenthesis.

**Table 5:** Local Crystal Structure Perturbation for Substituting a B- or N-Site with O^a^

bond	bond length (Å)					
	defect O_B_			defect O_N_		
	freestanding	HOPG	Ni(111)	freestanding	HOPG	Ni(111)
B–B/N–N	2.78	2.71	2.59	2.59	2.56	2.45
B/N–O	1.61	1.56	2.02	1.50	1.48	1.51
(B–N)_1_	1.42	1.41	1.41	1.42	1.41	1.46
(B–N)_2_	1.45	1.43	1.45	1.46	1.45	1.44
(B–B/N–N)_bulk_	2.51	2.49	2.49	2.51	2.49	2.49
(B–N)_bulk_	1.45	1.44	1.44	1.45	1.44	1.44

a (B–N)_1_ corresponds to the B–N bond lengths in the first coordination shell and (B–N)_2_ in the second coordination shell, as illustrated in Figure 6a,b. The bulk B–B, N–N, and B–N bond lengths are given for comparison.

**Figure 7:**
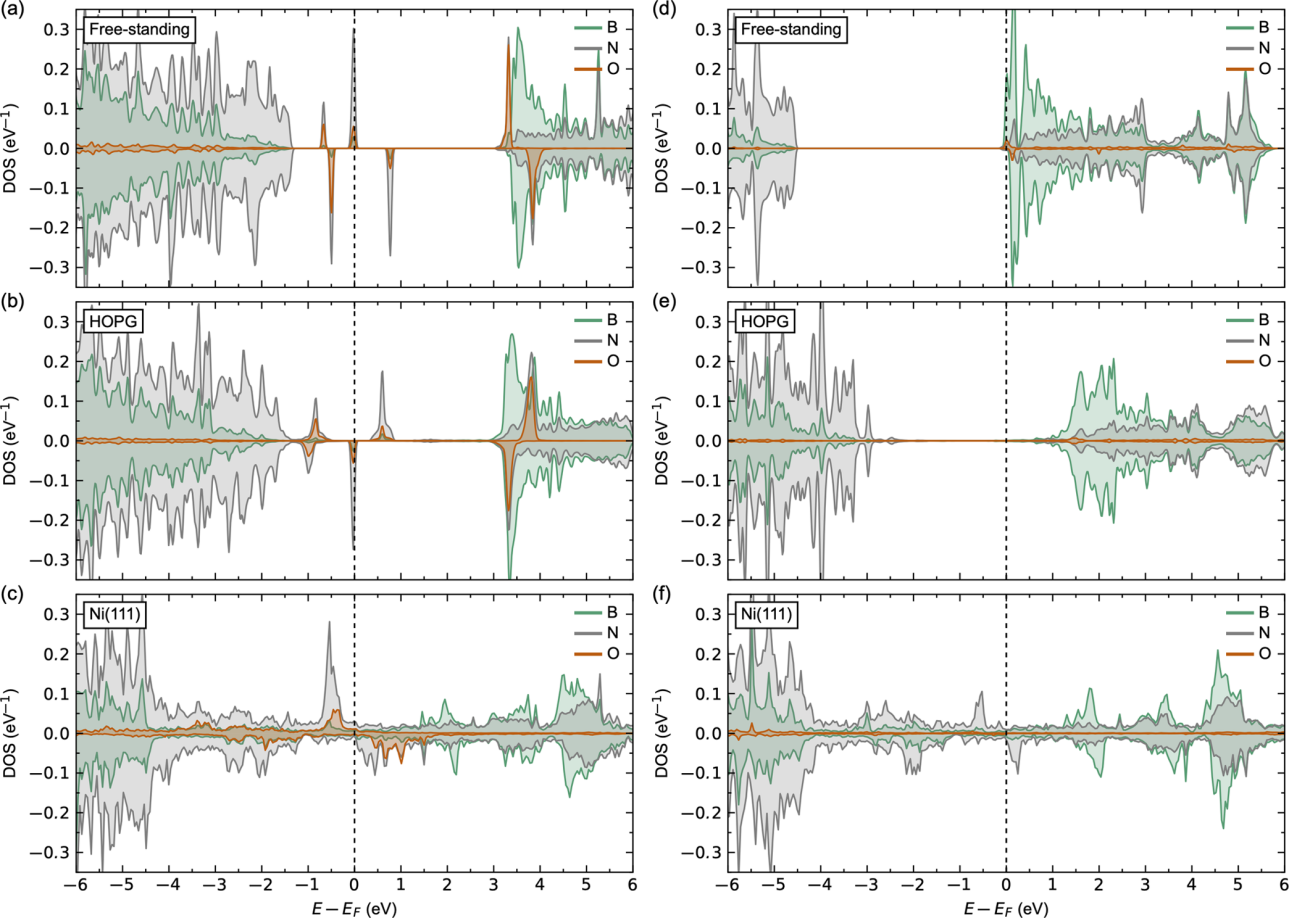
Calculated LDOS for (a–c) O_B_ and (d–f) O_N_ in (a,d) freestanding h-BN monolayer, (b,e) 1 ML of h-BN on HOPG, and (c,f) 1 ML of h-BN on Ni(111).

The LDOSs for h-BN with O_B_ or O_N_ are shown in Figure 7a–c and d–f, respectively, comparable to those reported in the literature.^94^ Oxygen is effectively an acceptor dopant when substituting boron, as can be seen from the calculated LDOS for O_B_ in freestanding h-BN in Figure 7a. We observe three occupied states in the band gap with mainly N 2p and O 2p character. Three donated electrons are localized on the three nitrogen atoms surrounding O_B_, apparent from induced N magnetic moments of 0.223–0.225 μ_B_. The oxygen shows a magnetic moment of 1.021 μ_B_. Qualitatively, a similar LDOS is observed for O_B_ in h-BN on HOPG in Figure 7b, with comparable N and O magnetic moments of 0.211–0.213 and 0.090 μ_B_, respectively. We observe no qualitative changes in the LDOS for O_B_ in h-BN on Ni(111) in Figure 7c, compared to pristine h-BN on Ni(111). The nitrogen atoms surrounding the defect show magnetic moments of 0.211–0.225 μ_B_, and the nickel atoms below the defect show reduced magnetic moments of 0.561–0.576 μ_B_. Oxygen shows a magnetic moment of 1.019 μ_B_.

Oxygen is a single-donor dopant when it substitutes nitrogen, as apparent from the calculated LDOS for O_N_ in the freestanding h-BN sheet in Figure 7d. The Fermi level is pinned in the conduction band and in particular on the occupied B 2p states, supporting the experimental finding that the electrons are the dominant free carriers, in contrast to the “p-type” character of pristine h-BN.^98,99^ These findings also support the experimentally found ∼100-fold lower electrical resistance,^15^ as well as the band gap narrowing by the shallow donor character of O_N_.^100^ For O_N_ in h-BN on HOPG in Figure 7e, the donated electrons occupy the intrinsically unoccupied states within the band gap from the weak film/substrate electronic coupling described above. No significant changes in magnetic moments are observed for O_N_ in the freestanding monolayer and HOPG systems. Finally, we observe no qualitative changes in the LDOS for O_N_ in h-BN on Ni(111) (Figure 7f) as compared to pristine h-BN on Ni(111). The electrons from the oxygen are donated to the nickel situated beneath the oxygen site, apparent from the significant decrease in the Ni magnetic moment of 0.297 μ_B_. No significant changes in B, N, and O magnetic moments are observed.

The changes in total magnetization with O_B_ relative to pristine h-BN are 0.817, 0.813, and 1.995 μ_B_ for the freestanding monolayer, h-BN/HOPG, and h-BN/Ni(111), respectively. The corresponding changes in total magnetization with O_N_ are 0.170 and −0.729 μ_B_ for the freestanding monolayer and h-BN/Ni(111), while no change in total magnetization is observed for h-BN/HOPG, as described above. As for the intrinsic defects described above, the absolute values of the defect-induced changes in total magnetizations in the freestanding monolayer are different from those reported in the literature.^94^

**Figure 8:**
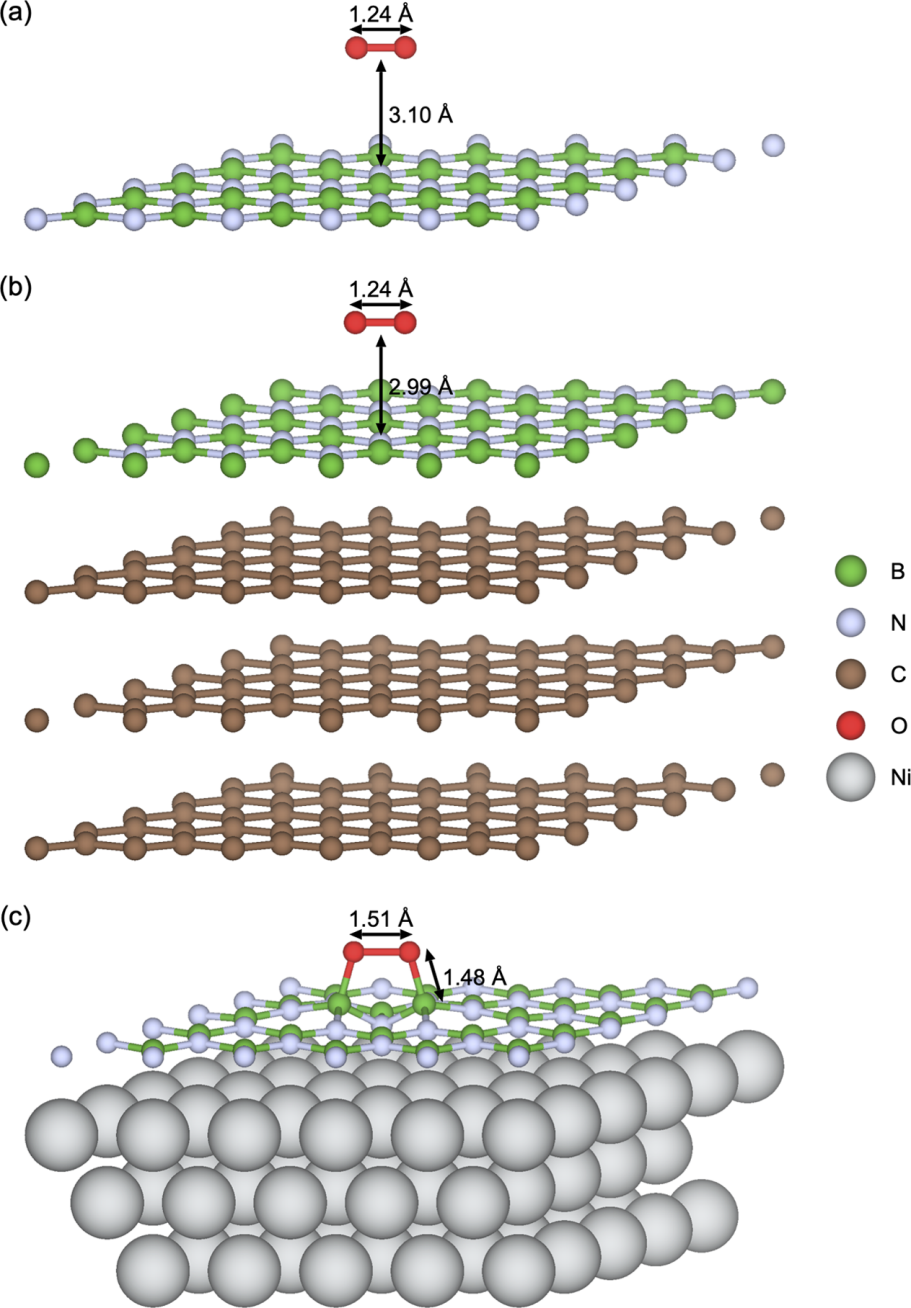
Side view of the calculated crystal structures for an O_2_ molecule adsorbed on top of (a) freestanding h-BN, (b) 1 ML of h-BN on HOPG, and (c) 1 ML of h-BN on Ni(111).

Note that while the h-BN_*x*_O_*y*_ bonding can also, in principle, be due to oxygen adsorption on the surface, oxygen adsorption is unlikely to occur since pristine h-BN is known to be inert toward oxidation. To corroborate the assumed inertness toward oxidation for pristine h-BN, we calculated the oxygen adsorption on pristine h-BN surfaces for the three different systems investigated. Figure 8 shows the DFT relaxed supercells for an O_2_ molecule on (a) a freestanding monolayer of h-BN, (b) 1 ML of h-BN on HOPG, and (c) 1 ML of h-BN on Ni(111). Here, we assume that the oxygen atoms will preferably bond with two nearest B atoms on the surface,^67−71^ where all structures were initialized with a B–O bond length of 1.4 Å.

After structural relaxation, the O_2_ molecule detaches from the freestanding monolayer (Figure 8a) and 1 ML h-BN/HOPG (Figure 8b) surfaces, which becomes apparent from the resulting distances between O_2_ and h-BN of, respectively, 3.10 and 2.99 Å, and bulk like O–O bond lengths of 1.24 Å. The O_2_ molecule will, however, adsorb on 1 ML h-BN on the Ni(111) surface (Figure 8c). The resulting B–O bond lengths after structural relaxation are 1.48 Å, where the two B atoms bonding to O are protruding out from the surface. An elongated O–O bond length of 1.51 Å is observed. These structural properties are in good agreement with a previous work.^67−71^ The binding energies for the O_2_ molecule on the freestanding monolayer, 1 ML h-BN/HOPG, and h-BN/Ni(111) surfaces are, respectively, −0.10, −0.13, and −2.23 eV, which suggest a strong interaction between O_2_ and h-BN/Ni(111) and weak interactions with freestanding h-BN and h-BN/HOPG, in line with the structural properties mentioned above and in previous studies.^67−71^ This further supports a low reactivity toward oxidation on freestanding h-BN and on h-BN/HOPG surfaces and high reactivity toward oxidation on the h-BN/Ni(111) surface. Since h-BN is shown to be decoupled from the substrate for multilayer thick films, these results suggest that any observed h-BN_*x*_O_*y*_ bonding for deposited h-BN films that are thicker than one monolayer cannot be explained by oxygen adsorption on the surface.

### 3.5 Carbon Defect Chemistry

Finally, we address the carbon defect chemistry with respect to the choice of the substrate, focusing first on in-plane carbon defects. The formation energies for carbon substitution on the boron (C_B_) and nitrogen (C_N_) sites are summarized in Table 2.

The formation energy of C_B_ is found to be ∼1–3 eV lower than for C_N_ for all systems investigated. The formation energies in the freestanding monolayer are comparable to those reported in bulk h-BN in N-rich conditions of 1.75 and 4.25 eV, respectively.^92^ A major difference between C_B_ and C_N_ defects in charge-neutral cells is that the former is an electron donor, while the latter acts as an electron acceptor, Figure 10a,d, respectively. From an electronic point of view, the acceptor C_N_ costs less energy than the donor C_B_, which induces occupation of a band gap state ∼3.4 eV above the valence band maximum. The comparison between the defect formation energies of the freestanding, wide band gap, semiconducting h-BN, and metallic h-BN on Ni(111) conclude that the metallic character reduces to half of its semiconducting energy values. As expected, the semiconducting h-BN on HOPG systems exhibit almost the same formation energy with the freestanding h-BN for the C_N_ defect; however, the formation energy for the C_B_ defect in h-BN on HOPG systems is less than half that of the freestanding h-BN case. It seems that the extra electron of the boron substitution with carbon is much more active on HOPG, and a similar effect can be seen in the Ni(111) case, compared to the more “inert” free standing h-BN. The energy preference for C_B_ over C_N_ can be explained by the associated structural screening, as described further below. The calculated formation energies for C_B_ and C_N_ are significantly lower than the corresponding intrinsic defects in Table 2. This means that the inherent point defects present after synthesis will likely be filled by any carbon impurity present in the deposition chamber, *e.g.*, from carbon residue in silver paste used for mounting BN targets for PLD.

**Figure 9:**
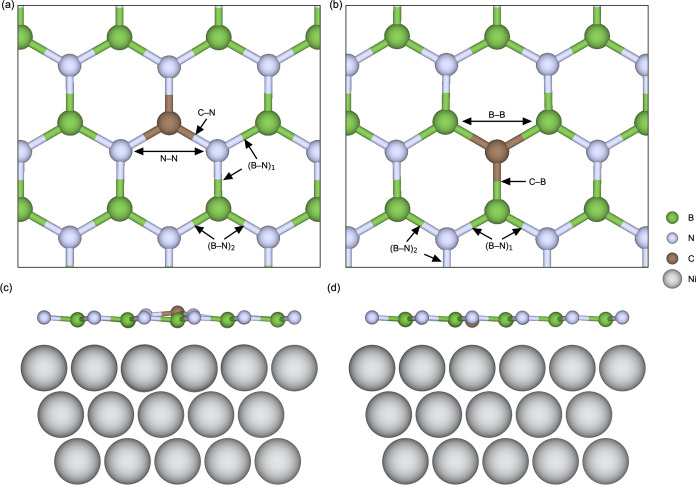
Top view of the local crystal structure in the vicinity of (a) C_B_ and (b) C_N_ in freestanding h-BN. Side view of (c) C_B_ and (d) C_N_ in 1 ML h-BN on Ni(111).

The structural screening for C_B_ and C_N_ is summarized by the local bond lengths in Table 6 and illustrated for the freestanding monolayer in Figure 9a,b (all C_B_ and C_N_ structures are visualized in Figures S13 and S14). The defect geometries in the freestanding monolayer are comparable to those reported in the literature.^93,96^ C_B_ induces a small local contraction, as apparent from the shortened N–C bond lengths of 1.41, 1.37, and 1.37 Å for the freestanding, HOPG, and Ni(111) systems, respectively, compared to the bulk values of 1.44–1.45 Å. The local contraction is further confirmed by the reduced N–N distances of, respectively, 2.44, 2.37, and 2.36 Å, compared to the bulk values of 2.49–2.51 Å. A small expansion of 0.01–0.03 Å is observed for the first coordination shell, while no significant perturbations are observed for the second coordination shell. The carbon atom in h-BN on Ni(111) is slightly protruding out of the h-BN layer toward the vacuum region, illustrated in Figure 9c. C_N_, on the other hand, induces a local structural expansion, apparent from the increased B–C bond lengths of 1.51, 1.48, and 1.47 Å and increased B–B distances in the vicinity of the defect of 2.61, 2.56, and 2.54 Å for freestanding h-BN, h-BN on HOPG, and h-BN on Ni(111), respectively. No significant perturbations are observed for the first and second coordination shells. The stability of C_B_ compared to C_N_ can thus be explained by the associated structural screening, where structures with C_N_ will have to accommodate large local stresses caused by the expanded B–C bonds. This observation is important when discussing in-plane h-BCN hybrid structures, as elaborated below.

**Table 6:** Local Crystal Structure Perturbation for Substituting a B- or N-Site with C^a^

bond	bond length (Å)					
	defect C_B_			defect C_N_		
	freestanding	HOPG	Ni(111)	freestanding	HOPG	Ni(111)
B–B/N–N	2.44	2.37	2.36	2.61	2.56	2.54
B/N–C	1.41	1.37	1.37	1.51	1.48	1.47
(B–N)_1_	1.46	1.47	1.47	1.44	1.44	1.44
(B–N)_2_	1.45	1.43	1.44	1.45	1.43	1.43
(B–B/N–N)_bulk_	2.51	2.49	2.49	2.51	2.49	2.49
(B–N)_bulk_	1.45	1.44	1.44	1.45	1.44	1.44

a (B–N)_1_ corresponds to the B–N bond lengths in the first and (B–N)_2_ in the second coordination shell, as illustrated in Figure 9a,b. The bulk B–N bond lengths are given for comparison.

The binding heights for C_B_ and C_N_ for HOPG and Ni(111), summarized in Table 4, are comparable to those for pristine h-BN. The binding heights for the protruding boron or nitrogen atoms for h-BN on Ni(111) in Figure 9c,d are marked in parenthesis.

**Figure 10:**
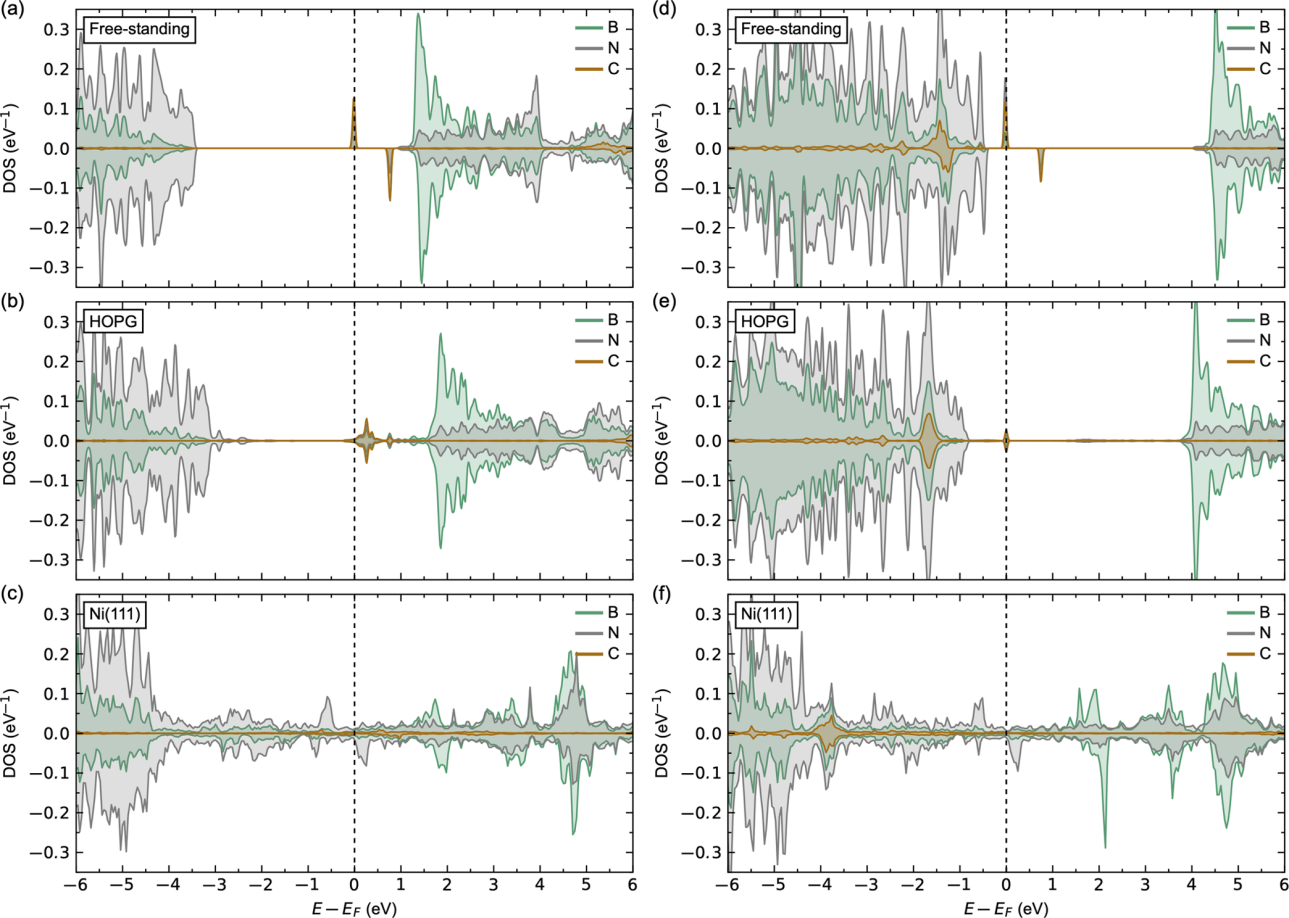
Calculated LDOSs for (a–c) C_B_ and (d–f) C_N_ in (a,d) freestanding h-BN monolayer, (b,e) 1 ML of h-BN on HOPG, and (c,f) 1 ML of h-BN on Ni(111).

The LDOSs for C_B_ and C_N_ are shown in Figure 10a–c and d–f, respectively. Substitution with carbon results in one occupied and one unoccupied defect state in the band gap , as shown for the LDOS in freestanding h-BN in Figure 10a,d, in agreement with previous studies.^93,94,96^ Note that the occupied defect states are completely filled. The apparent crossing of the Fermi level at the defect state is due to the applied Gaussian broadening to the DOS plot. The defect states are close to the CBM or VBM (valence band maximum) for C_B_ and C_N_, respectively, and is of mainly C 2p character. This is also apparent from the resulting magnetic moments, where C_B_ and C_N_ show magnetic moments of 0.330 and 0.274 μ_B_, respectively, with no significant changes in the h-BN magnetic. Comparable LDOSs are obtained for C_B_ and C_N_ in h-BN on HOPG in Figure 10b,e, respectively, however without any induced magnetism. Similar to the previous defects, we observe no qualitative changes in the LDOS with C_B_ or C_N_ in h-BN on Ni(111) compared to that for pristine h-BN, where C_B_ and C_N_ show magnetic moments of 0.028 and 0.006 μ_B_, respectively. No significant changes in magnetic moments for B and N are observed.

The changes in total magnetization with C_B_ relative to pristine h-BN are 0.550 and −0.496 μ_B_ for the freestanding monolayer and h-BN/Ni(111), respectively. The corresponding change in total magnetization with C_N_ are 0.511 and −0.441 μ_B_ for the freestanding monolayer and h-BN/Ni(111), respectively. No change in magnetism is observed for h-BN/HOPG, as described above. The defect-induced changes in total magnetizations in the freestanding monolayer are half of those reported previously.^94^

**Figure 11:**
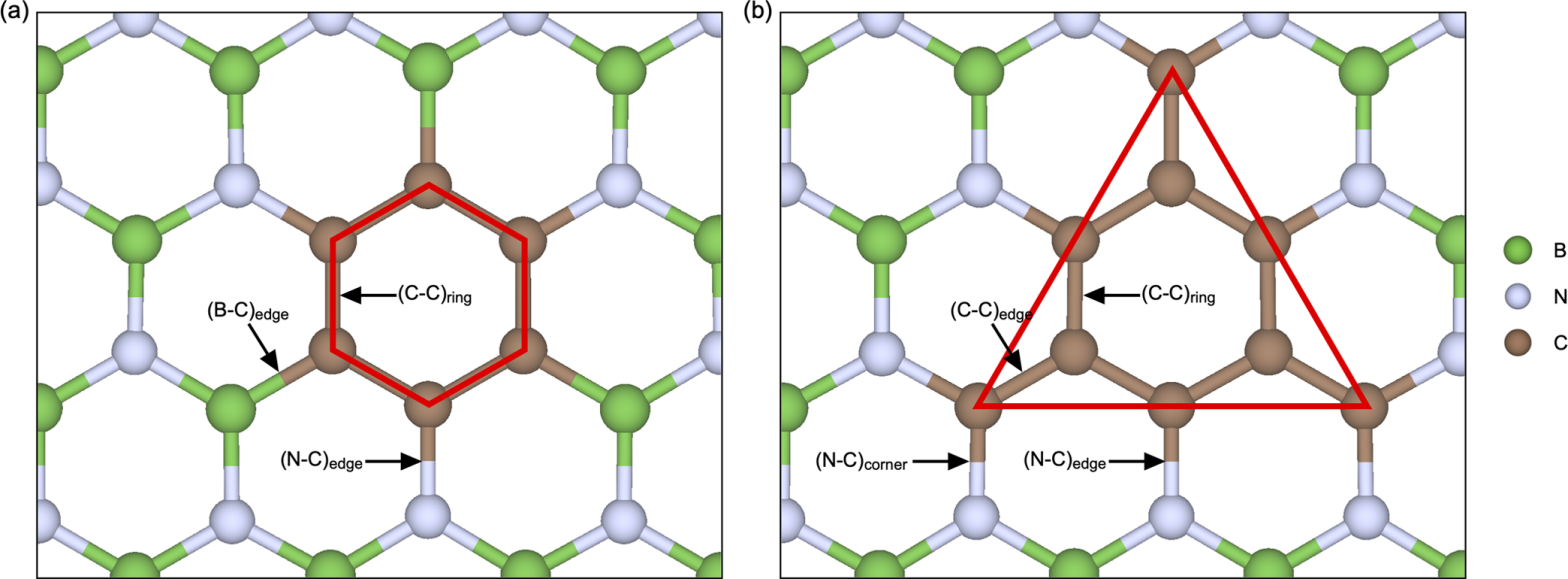
Top view of the two h-BCN configurations investigated: (a) hexagonal-shaped graphene sheet embedded in h-BN (“C6”) and (b) nitrogen-terminated triangular-shaped graphene sheet embedded in h-BN (“C9”). Red solid lines illustrate the shape of the graphene fragments.

Next, we investigate more experimentally relevant in-plane h-BN/graphene hybrid structures, referred to as h-BCN in the following. Two different in-plane h-BCN configurations have been investigated. The first configuration labeled “C6” (Figure 11a), is a hexagonal shaped graphene sheet consisting of six carbon atoms embedded in the h-BN layer. The graphene sheet is bonded to an equal number of B and N atoms. The second configuration labeled “C9” (Figure 11b), is a triangular shaped graphene sheet consisting of nine carbon atoms embedded in the h-BN layer. The graphene sheet is here only bonded to N, however, note that both nitrogen and boron bonded triangular shapes are possible.^31^ However, as described above and further elaborated below, B–C bonding comes with large tensile stress. Furthermore, preliminary in-house XPS results show no significant B–C bonding for PLD deposited h-BN films with C signature. This is also supported by the calculated formation energy of C_B_ being lower than for C_N_, in agreement with the literature,^56^ and hence, the N–C bonding is believed to be favorable compared to B–C bonding. We therefore focus mainly on N–C-bonded triangular shapes in the following.

The resulting bond lengths in the vicinity of the two h-BCN structures for freestanding h-BCN and on top of the two substrates are summarized in Table 7. In the nitrogen-only terminated “C9” structures, only subtle local crystal structure perturbations are observed. The most significant local structure perturbations are found in the “C6” structure, where the B–C bonds are significantly longer than the N–C and B–N bonds. These results suggest that B–C bonding in the h-BCN hybrid structures is not favored, as these structures will have to accommodate large tensile stress caused by the expanded B–C bonds. Comparable results are observed for larger triangular shapes (see Supporting Note 4).

**Table 7:** Calculated Bond Lengths for the Two Different In-Plane h-BCN Configurations with Respect to the Substrate^a^

bond	bond length (Å)		
	freestanding	HOPG	Ni(111)
C6			
(C–C)_ring_	1.40	1.41	1.41
(N–C)_edge_	1.42	1.42	1.40
(B–C)_edge_	1.50	1.50	1.49
C9			
(C–C)_ring_	1.43	1.43	1.43
(C–C)_edge_	1.41	1.42	1.43
(N–C)_edge_	1.42	1.41	1.38
(N–C)_corner_	1.40	1.40	1.37
Bulk			
(B–N)_bulk_	1.45	1.44	1.44

a Here, “ring”, “edge”, and “corner” refer to the chemical bonds in the (constituting) carbon ring, at the edge of the ring, and at the corners of the triangular shape, respectively, illustrated in Figure 11. Bulk B–N bulk lengths are shown for comparison.

**Figure 12:**
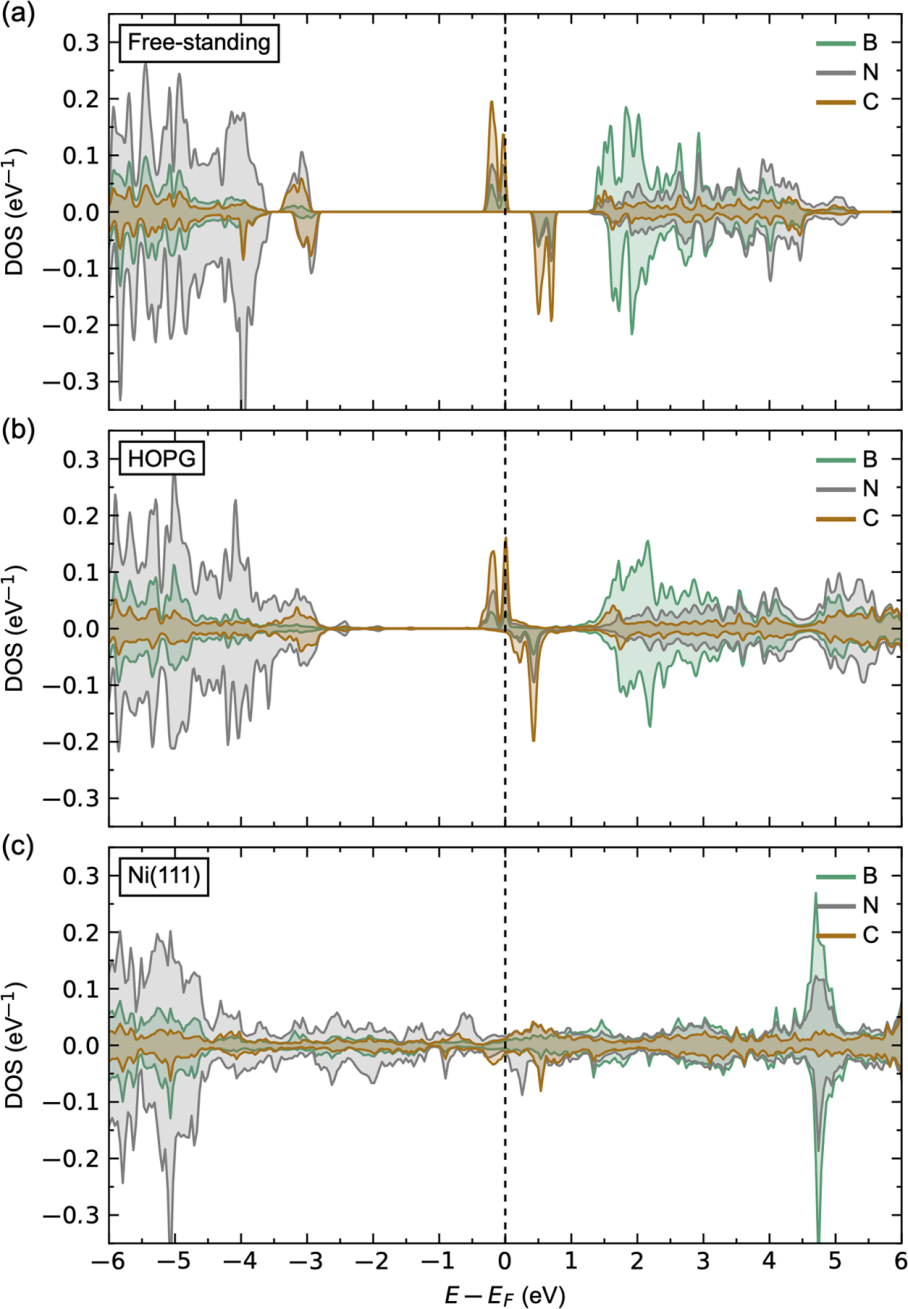
Calculated LDOS of “C9” h-BCN hybrid structures for (a) freestanding h-BCN monolayer, (b) 1 ML of h-BCN on HOPG, and (c) 1 ML of h-BCN on Ni(111).

A comparison of the LDOS for the ”C9” configuration in a freestanding h-BCN sheet and on top of the two substrates is shown in Figure 12. The embedded graphene structure in the freestanding h-BCN sheet and on top of HOPG in Figure 12a,b, respectively, gives rise to both occupied and unoccupied defect states within the band gap. The substitution of boron by carbon donates electrons to the system, while the substitution of nitrogen by carbon donates holes to the system. Hence, in the N-terminated “C9” structure with six boron and three nitrogen substituted with carbon, the net sum will be an electron-doped system, where the Fermi level is pinned in the h-BN band gap on occupied C-dominated levels.^31^ The embedding of graphene in these two systems also induces weak magnetism. The induced magnetic moments retain the initialized ferromagnetic ordering, apparent from the LDOS in Figure 12. The hybrid h-BCN structure on Ni(111) shows significantly different electronic DOS (Figure 12c) similar to pristine h-BN on Ni(111) described above, which we attribute to significant out-of-plane orbital overlap between the h-BCN layer and the nickel substrate.

Due to the similar structures of h-BN and graphene, out-of-plane stacking of h-BN and graphene is expected to be experimentally relevant. To provide some insight into the expected surface reactivity of such out-of-plane hybrid structures, we have performed calculations on a selected series of different stacking sequences of 2 ML of h-BN and/or graphene on HOPG and Ni(111) (see Supporting Note 6). The results show that the exposed surface becomes electronically decoupled from the substrate after the first monolayers, in line with the multilayer results in Figures S4 and S5. Whence, out-of-plane 2D vdW heterostructure surfaces are expected to behave qualitatively similar to their monolayer counterparts.

## 4 Conclusions

The electronic properties and defect chemistry of pure h-BN and defect h-BN films with respect to the type of substrate were addressed by DFT calculations. h-BN is HOPG tolerant, *i.e.*, the electronic properties for freestanding h-BN monolayers and h-BN on top of HOPG are nearly identical. Ni(111) as a substrate, on the other hand, completely suppresses the electronic band gap and renders monolayer h-BN electronically conducting. This different behavior influences the predicted defect formation energies, where we find significantly lower formation energies for intrinsic vacancies and oxygen and carbon substitutional defects in h-BN on Ni(111). Hence, deposited films are expected to contain such point defects. As we find that the formation energies for the substitutional defects are significantly lower than those for the corresponding intrinsic defects regardless of the substrate, vacancies formed during deposition are prone to be filled either by oxygen or carbon present in the deposition chamber or upon air exposure.

In-plane h-BCN hybrid structures are expected to be terminated mainly by N–C bonding, which is attributed to the lower formation energy of C_B_ with respect to C_N_ due to the large tensile strain associated with B–C bonding, and consequently, the N–C bonding governing the bond formation in carbon-doped h-BN. Electronic properties of the C-doped h-BN, establish an n-type character of the C_B_ defect in contrast to the p-type character of the C_N_. Hence, the energetically favorable C_B_ defect imposes the n-type semiconductivity in C-doped h-BN. Considering the larger difference in formation energy between C_N_ and C_B_, comparing the freestanding h-BN and on HOPG, the n-type character of C-doped h-BN should increase significantly on HOPG.

The h-BN surface becomes electronically decoupled from the substrate when exceeding a monolayer thickness, implying that the surface electronic properties and defect chemistry for multilayer h-BN films should be comparable to that of a freestanding h-BN layer. This is also observed for stacked out-of-plane h-BN/graphene hybrid structures.

Finally, the calculated electronic structures suggest that O_N_ is the dominant reason for unintentional n-type doping of h-BN, which supports experimentally identified lower electrical resistance and band gap narrowing by the shallow donor character of O_N_.^15,100^

## Supporting Information Available

Additional details for the vdW functional testing and configuration- and size-dependent studies of the h-BN hybrid structures (PDF)
