# Supplementary material for: Electronic Structure and Surface Chemistry of Hexagonal Boron Nitride on HOPG and Nickel Substrates
Source: ACS Omega. 2023 Jul 5;8(28):24813–30. doi: 10.1021/acsomega.3c00562 (PMC10357548; doi:10.1021/acsomega.3c00562)
Supplement: Supplementary file 1 — ao3c00562_si_001.pdf [file ao3c00562_si_001.pdf]

Supplementary Information

Electronic structure and surface chemistry of hexagonal boron nitride on HOPG and nickel substrates

Didrik René Småbråten[a,b,†], Inger-Emma Nylund[a], Kenneth Marshall[a], Julian Walker[a], Maria Benelmekki[a], Mari-Ann Einarsrud[a], Joseph Kioseoglou[b], and Sverre M. Selbach[a,*]

[a]*Department of Materials Science and Engineering, NTNU Norwegian University of Science and Technology, Trondheim, Norway*

[b]*Department of Physics, Aristotle University of Thessaloniki, Thessaloniki, Greece*

*E-mail: selbach@ntnu.no

Present address: †SINTEF Industry, Sustainable Energy Technology, Oslo, Norway.

Supplementary Note 1. Structural configurations of the heterostructures

The energetics for different structural configurations of h-BN on HOPG and Ni(111) were calculated with supercells consisting of three atomic layer thick slabs with a 20 Å vacuum spacing, as described in the main text. The B and N atoms can be positioned on three sites on the HOPG substrate relative to the atomic positions in the slab; sitting on top of an atom in the top layer which again sits on top of an atom in the second layer referred to as the "*tt*"-site, sitting on top of an atom in the top layer which again sits on top of a hollow site in the second layer referred to as "*th*"-site in the following, and sitting on top of a hollow site referred to as the "*h*"-site in the following. This gives six different h-BN/HOPG configurations, B_h-B_{th}, B_h-B_{tt}, B_{th}-N_h, B_{th}-N_{tt}, B_{tt}-N_h, B_{tt}-N_{th} (Figure S1). The calculated binding energies and binding heights for the six configurations are summarised in Table S1 The two configurations B_{th}-N_h and B_{tt}-N_h show similar binding energies within 0.06 meV/h-BN, and equal binding heights of 3.27 Å, in agreement with ref.[16]. No corrugation of h-BN is observed. The two configurations B_{th}-N_h and B_{tt}-N_h correspond to "A-A" and "A-B" stacking of h-BN relative to C, respectively, with B sitting on top of C and N on top of a hollow site. The energy difference of 0.06 meV/h-BN is within the energy resolution limit of the DFT calculations. The B_{tt}-N_h configuration was chosen, as this mimics the lowest energy "A-B" stacking of both graphite and h-BN.

As for the h-BN/HOPG system, the B and N atoms can be placed on three different sites on the Ni(111) surface relative to the Ni positions in the slab; sitting on top of the first atom layer referred to as the "*top*"-site, sitting on top of the second atom layer referred to as the "*hcp*"-site, or sitting on top of the third referred to as the "*fcc*"-site. With two atoms B and N, we have in total six different structural configurations for the h-BN/Ni(111) heterostructure, B_{fcc}-N_{hcp}, B_{fcc}-N_{top}, B_{hcp}-N_{fcc}, B_{hcp}-N_{top}, B_{top}-N_{fcc}, and B_{top}-N_{hcp} (Figure S2). The resulting binding energies and average binding heights for the six configurations are summarized in Table S6. The energetically most favored configuration is B_{fcc}-N_{top}, in agreement with previous studies.[17–26] This configuration has an binding height of 2.11 Å, where the B atom is closer to the surface than the N atom with a buckling of 0.10 Å.

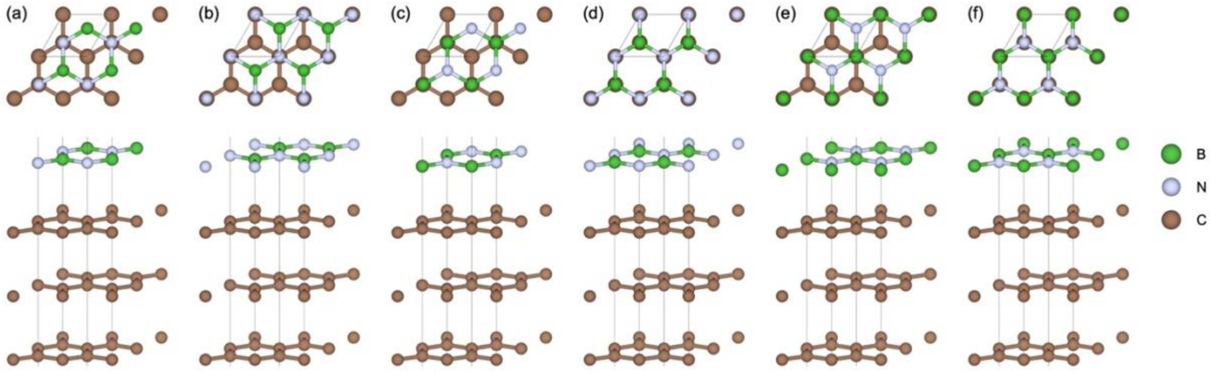

Figure S1. DFT relaxed h-BN/HOPG configurations investigated; (a) B_h-B_{th}, (b) B_h-B_{tt}, (c) B_{th}-N_h, (d) B_{th}-N_{tt}, (d) B_{tt}-N_h, (e) B_{tt}-N_{th}. The configuration in (e) corresponds to the model system used in the main text. The configurations are illustrated for a *p*(2x2) surface, where the *p*(1x1) boundaries are illustrated by the solid black lines.

Table S1. Calculated binding energies, E_b, and the binding heights, h, between the h-BN monolayer and the HOPG surface for the six different h-BN/HOPG configurations.

	E_b (meV/h-BN)	h (Å)
B_h-N_{th}	–51.09	3.44
B_h-N_{tt}	–51.28	3.43
B_{th}-N_h	–60.27	3.27
B_{th}-N_{tt}	–49.47	3.48
B_{tt}-N_h	–60.21	3.27
B_{tt}-N_{th}	–49.27	3.49

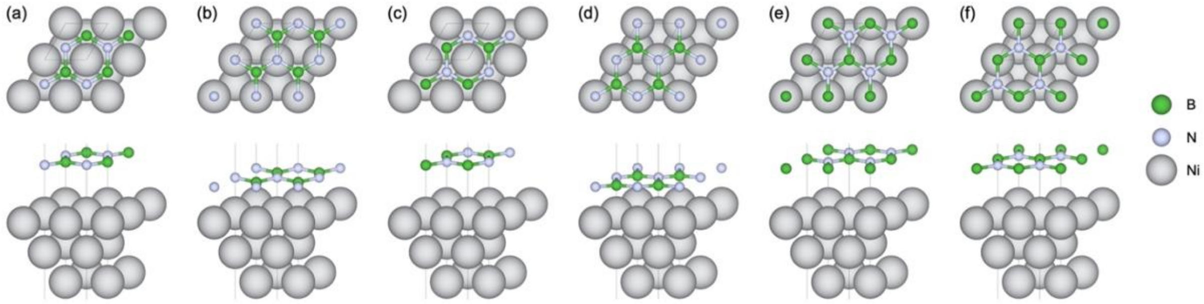

Figure S2. DFT relaxed h-BN/Ni(111) configurations investigated; (a) B_{fcc}-B_{hcp}, (b) B_{fcc}-B_{top}, (c) B_{hcp}-N_{fcc}, (d) B_{hcp}-N_{top}, (d) B_{top}-N_{fcc}, (e) B_{top}-N_{hcp}. The configuration in (b) corresponds to the model system used in the main text. The configurations are illustrated for a *p*(2x2) surface, where the *p*(1x1) boundaries are illustrated by the solid black lines.

Table S2. Calculated binding energies, E_b, and the binding heights, h, between the h-BN monolayer and the Ni(111) surface for the six different h-BN/Ni(111) configurations.

	E_b (meV/h-BN)	h (Å)
B_{fcc}-N_{hcp}	–57.80	3.33
B_{fcc}-N_{top}	–101.41	2.11
B_{hcp}-N_{fcc}	–58.63	3.34
B_{hcp}-N_{top}	–96.61	2.11
B_{top}-N_{fcc}	–59.13	3.33
B_{top}-N_{hcp}	–59.22	3.33

Supplementary Note 2. Assessment of the vdW functionals

The experimentally reported and calculated lattice parameters and binding energies of h-BN in the two stable polymorphs ($P\bar{6}m_2$ and $P6_3/mmc$) and of graphite ($P6_3/mmc$) are summarized in Table S3 to Table S5, using the seven different vdW functionals[1–6], the PBEsol functional[7], and the standard PBE[8] and LDA[9] functionals pseudopotentials. Computational details are described in the main text. A comparison between the calculated and the experimental bulk lattice parameters of h-BN, graphite, and Ni using the rev-vdW-DF2 functional are given in Table S6.

Table S3. Calculated lattice parameters and interlayer binding energies of h-BN ($P\bar{6}m_2$) calculated using DFT, including deviations from the experimental values. The binding energies were calculated assuming the experimental a lattice parameter.

	a (Å)	Δ (%)	c (Å)	Δ (%)	E_b (meV/atom)	E_g (eV)	Δ (%)
LDA	2.490	–0.6	6.393	–4.0	–28	4.22	–29.3
PBE	2.511	+0.3	7.868	+18.1	–2	4.52	–24.2
PBEsol	2.504	+0.0	6.824	+2.4	–6	4.33	–27.4
vdW-DF	2.523	+0.7	7.016	+5.3	–52	4.51	–24.4
vdW-DF2	2.521	+0.7	6.910	+3.7	–50	4.44	–25.7
rev-vdW-DF2	2.510	+0.2	6.476	–2.8	–58	4.32	–27.6
vdW-optB86b	2.511	+0.3	6.443	–3.3	–70	4.34	–27.3
vdW-optB88	2.510	+0.2	6.521	–2.1	–70	4.37	–26.8
vdW-optPBE	2.516	+0.5	6.709	+0.7	–63	4.42	–26.0
SCAN+rVV10	2.496	–0.3	6.365	–4.4	–53	4.84	–18.9
Exp.	2.504[10]	+0.0	6.661[10]	+0.0	–	5.97[11,12]	+0.0

Table S4. Calculated lattice parameters and interlayer binding energies of h-BN ($P6_3/mmc$) calculated using DFT, including deviations from the experimental values. The binding energies were calculated assuming the experimental *a* lattice parameter.

	a (Å)	Δ (%)	*c* (Å)	Δ (%)	E_b (meV/atom)	E_g (eV)	Δ (%)
LDA	2.490	–0.5	6.426	–3.5	–27	4.06	–32.0
PBE	2.511	+0.3	7.869	+18.1	–2	4.54	–24.0
PBEsol	2.504	+0.0	6.868	+3.1	–5	4.27	–28.4
vdW-DF	2.523	+0.8	7.029	+5.5	–52	4.48	–25.0
vdW-DF2	2.521	+0.7	6.918	+3.9	–50	4.38	–26.6
rev-vdW-DF2	2.510	+0.3	6.499	–2.4	–57	4.19	–29.8
vdW-optB86b	2.511	+0.3	6.468	–2.9	–70	4.21	–29.4
vdW-optB88	2.510	+0.2	6.540	–1.8	–69	4.26	–28.7
vdW-optPBE	2.516	+0.5	6.726	+1.0	–63	4.34	–27.3
SCAN+rVV10	2.496	–0.3	6.382	–4.2	–53	4.71	–21.1
Exp.	2.504[10]	0	6.661[10]	0	–	5.97[11,12]	0

Table S5. Lattice parameters and interlayer binding energies of graphite ($P6_3/mmc$) calculated using DFT, including deviations from the experimental values. The binding energies were calculated assuming the experimental *a* lattice parameter.

	a (Å)	Δ (%)	*c* (Å)	Δ (%)	E_b (meV/atom)
LDA	2.446	–0.7	6.593	–1.8	–25
PBE	2.446	+0.1	8.108	+20.8	–1
PBEsol	2.459	–0.2	7.113	+6.0	–4
vdW-DF	2.477	+0.5	7.111	+6.0	–54
vdW-DF2	2.473	+0.4	6.971	+3.9	–52
rev-vdW-DF2	2.464	+0.0	6.603	–1.6	–59
vdW-optB86b	2.465	+0.1	6.592	–1.8	–71
vdW-optB88	2.464	+0.0	6.656	–0.8	–70
vdW-optPBE	2.470	+0.2	6.831	1.8	–64
SCAN+rVV10	2.449	–0.6	6.526	–2.8	–54
Exp.	2.464[13]	+0.0	6.711[13]	+0.0	25 – 57[14]

Table S6. Comparison between the calculated and the experimental bulk lattice parameters of h-BN, graphite, and Ni using the rev-vdW-DF2 functional.

	h-BN		Graphite		Ni
	a (Å)	*c* (Å)	*a* (Å)	*c* (Å)	*a* (Å)
DFT	2.510	6.476	2.464	6.603	3.494
Exp.	2.504[10]	6.661[10]	2.464[13]	6.711[13]	3.499[15]
Δ (%)	+0.2	–2.8	+0.0	–1.6	–0.1

Supplementary Note 3. h-BN film thickness dependency on the calculated structural and electronic properties

The h-BN film thickness dependency of the calculated structural and electronic properties for the two different substrates were calculated on supercells consisting of seven atomic layer thick metal slabs with a vacuum spacing of 40 Å, using the hard pseudopotentials supplied with VASP and the rev-vdW-DF2 functional.

Multiple monolayers (ML) of h-BN, up to a total of four, where added on top of the slabs. Representative calculated supercells of 4ML of h-BN on HOPG and Ni(111) are shown in Figure S3 and (b), respectively. The corresponding interlayer distances are summarized in Table S7 and Table S8, respectively. No significant changes in the interlayer distances are observed exceeding 2 ML of h-BN, indicating that the second layer of h-BN and all consecutive layers are decoupled from the substrate. This is further confirmed by the calculated local electronic density of states (LDOS) for 4ML of h-BN on HOPG (Figure S4(b)) and 4ML of h-BN on Ni(111) (Figure S5(b)), where all the h-BN monolayers after the first show LDOS comparable to that of the free-standing h-BN DOS in Figure 3(a) in the main text.

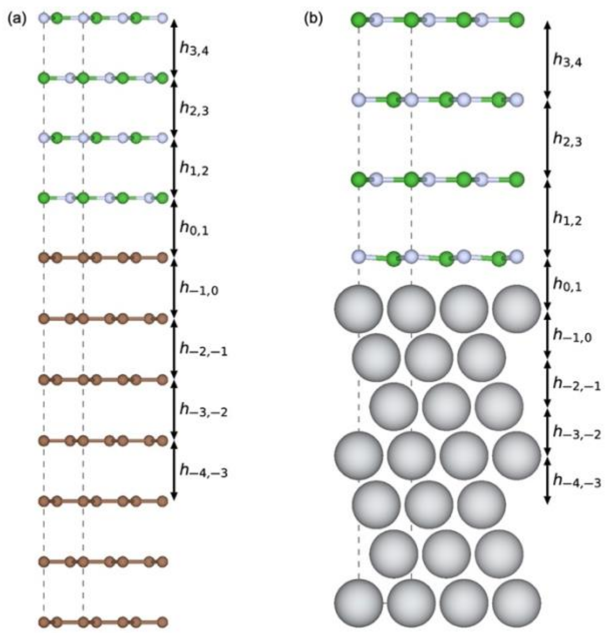

Figure S3. Crystal structures for DFT relaxed supercells of 4ML h-BN on (a) HOPG and (b) Ni(111), illustrating the interlayer distances *h* across the supercells. The interlayer distances are summarized in Table S7 and Table S8, respectively.

Table S7. Calculated interlayer distances, *h*, in the h-BN/HOPG system for increasing h-BN layer thickness. Here, subscript "0" corresponds to the substrate interfacial layer, subscript "1" the h-BN interfacial layer, and so forth, see Figure S3(a).

	Interlayer distance, *h* (Å)				
	Clean surface	1ML	2ML	3ML	4ML
$h_{-4,-3}$	3.30	3.30	3.30	3.30	3.30
$h_{-3,-2}$	3.32	3.33	3.33	3.33	3.33
$h_{-2,-1}$	3.32	3.33	3.33	3.33	3.33
$h_{-1,0}$	3.33	3.32	3.33	3.33	3.33
$h_{0,1}$		3.27	3.26	3.26	3.27
$h_{1,2}$			3.28	3.27	3.27
$h_{2,3}$				3.28	3.27
$h_{3,4}$					3.28

Table S8. Calculated interlayer distances, *h*, in the h-BN/Ni(111) system for increasing h-BN layer thickness. Here, subscript “0” corresponds to the substrate interfacial layer, subscript “1” the h-BN interfacial layer, and so forth, see Figure S3(b).

	Interlayer distance, *h* (Å)				
	Clean surface	1ML	2ML	3ML	4ML
$h_{-4,-3}$	2.02	2.02	2.02	2.02	2.02
$h_{-3,-2}$	2.02	2.01	2.01	2.01	2.01
$h_{-2,-1}$	2.02	2.01	2.01	2.01	2.01
$h_{-1,0}$	1.99	2.00	2.00	2.00	2.00
$h_{0,1}$		2.11	2.11	2.10	2.10
$h_{1,2}$			3.23	3.21	3.21
$h_{2,3}$				3.28	3.27
$h_{3,4}$					3.28

–2 eV to +2 eV).

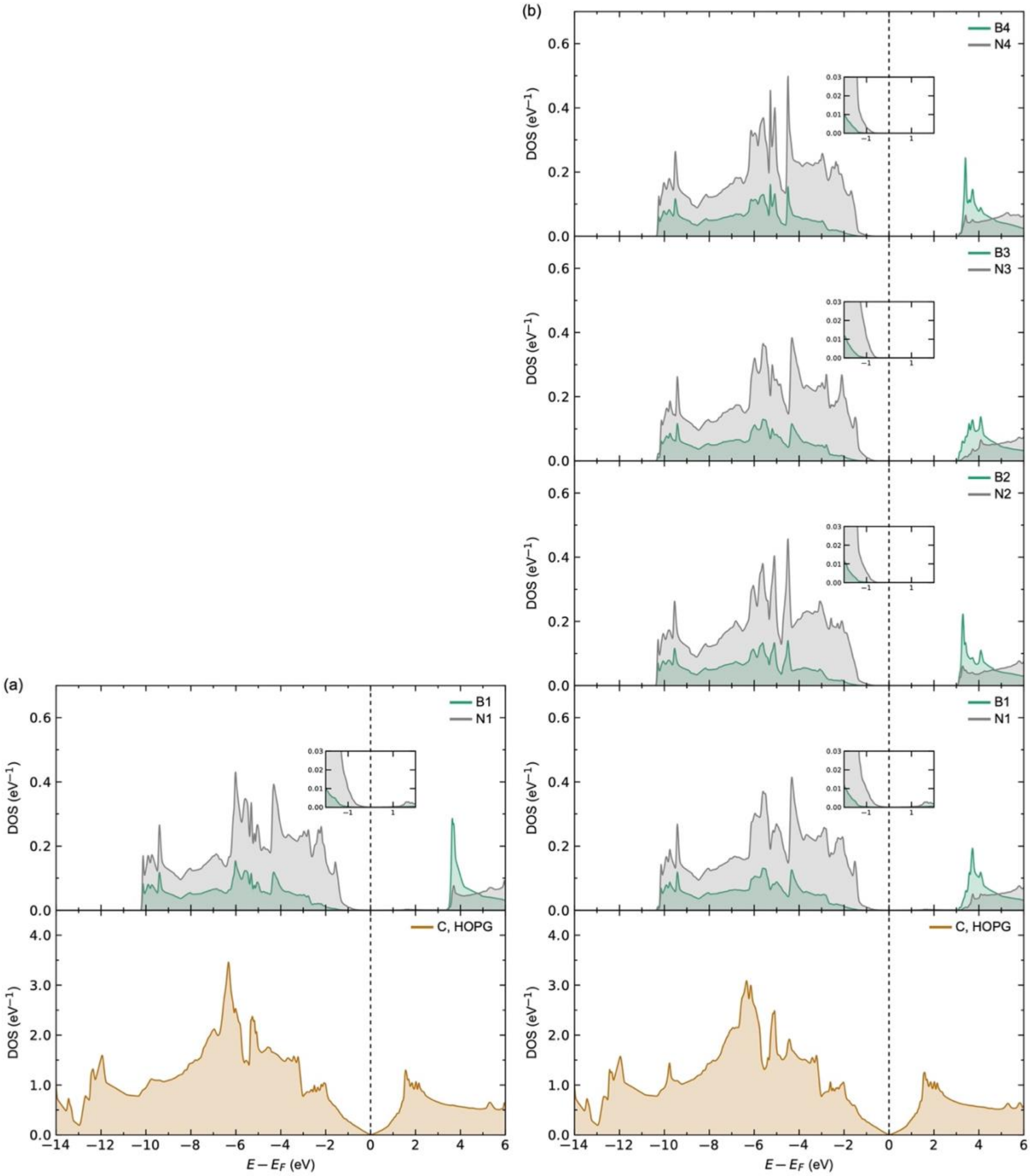

Figure S4. Calculated local electronic density of states (LDOS) for (a) 1ML and (b) 4ML of h-BN on HOPG. The numbers in (b) indicate the h-BN monolayer number starting from one closest to the substrate interface. The insets are zoomed in LDOS around the Fermi level (from –2 eV to +2 eV).

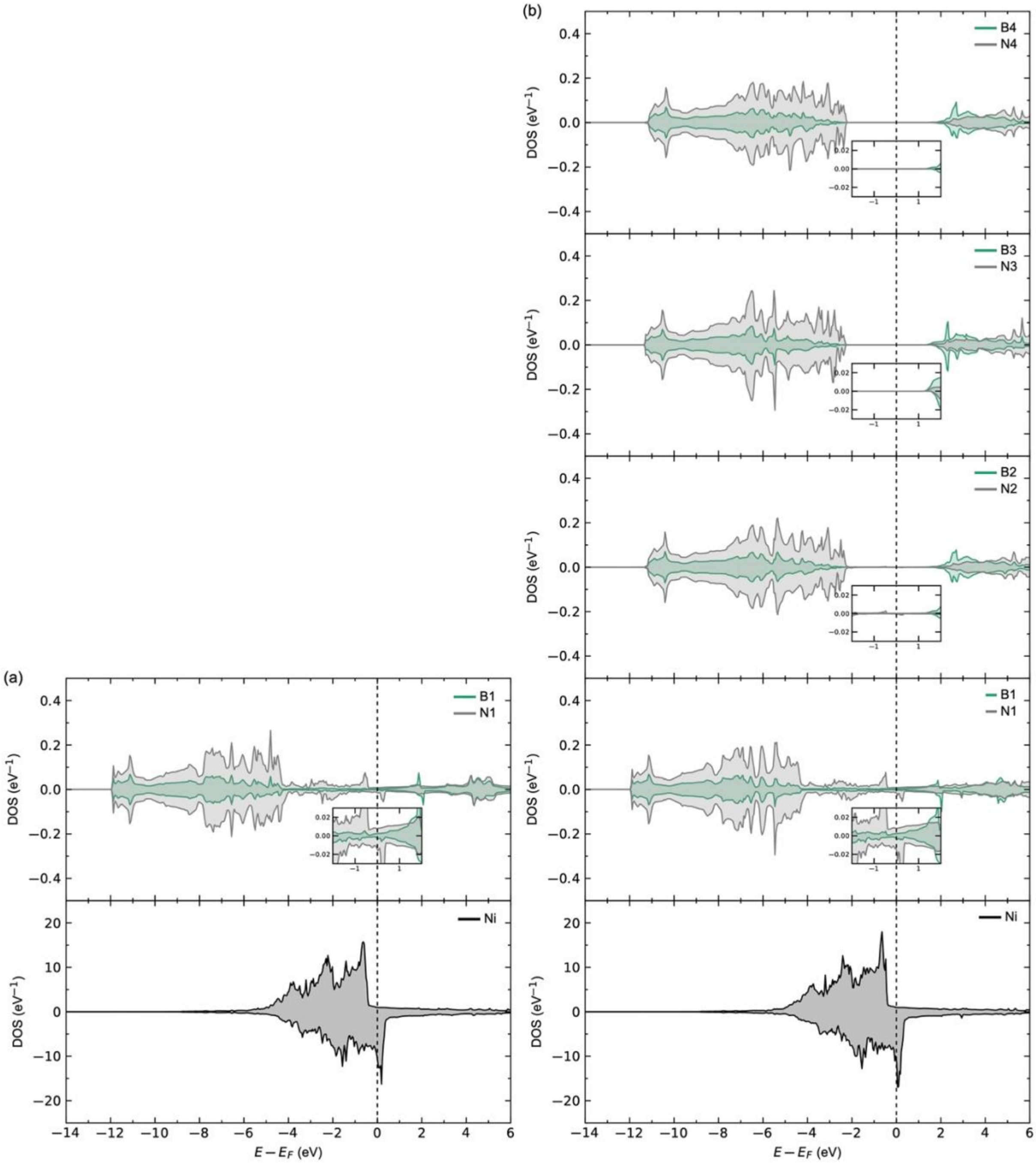

Figure S5. Calculated local electronic density of states (LDOS) for (a) 1ML and (b) 4ML of h-BN on Ni(111). The numbers in (b) indicate the h-BN monolayer number starting from one closest to the substrate interface. The insets are zoomed in LDOS around the Fermi level (from –2 eV to +2 eV).

Supplementary Note 4: Orbital resolved LDOS

The orbitally resolved LDOS for free-standing monolayer of pristine h-BN, 1ML of pristine h-BN on HOPG, and 1ML of pristine h-BN on Ni(111) are shown in Figure S6 to Figure S8, respectively.

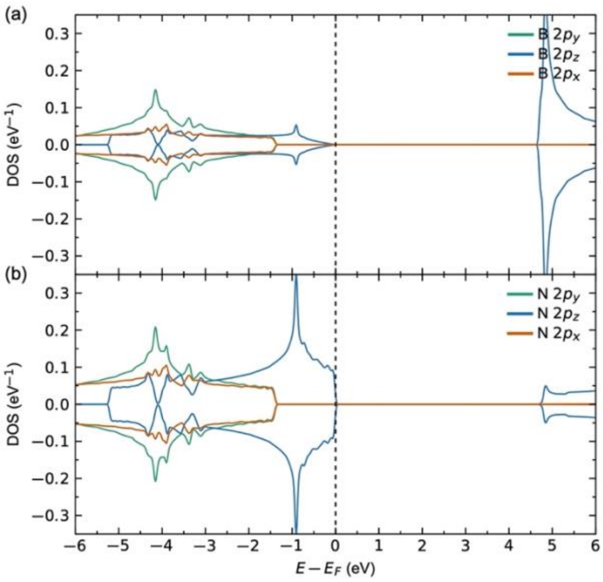

Figure S6. Orbital resolved LDOS for free-standing monolayer of pristine h-BN. (a) B 2*p* states, and (b) N 2*p* states.

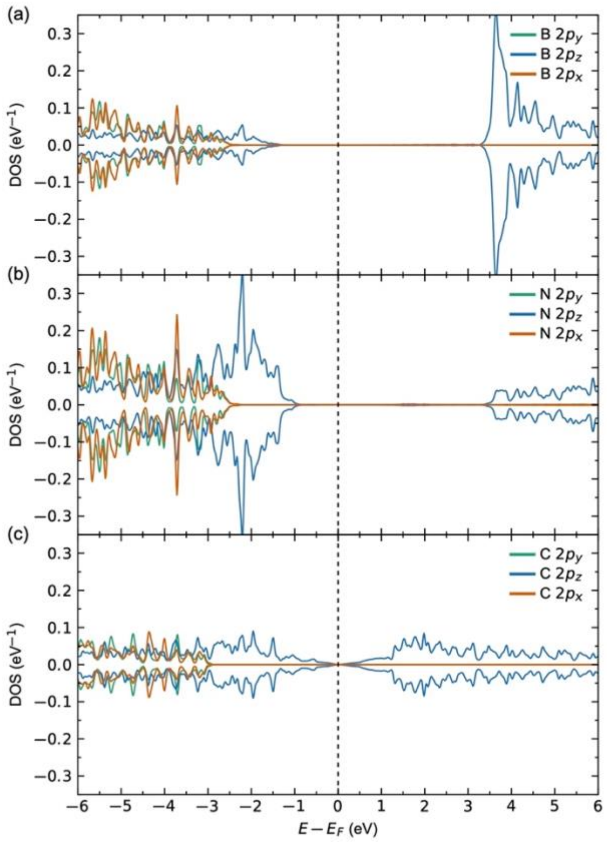

Figure S7. Orbital resolved LDOS for 1ML of pristine h-BN on HOPG. (a) B 2*p* states, (b) N 2*p* states, and (c) C 2*p* states.

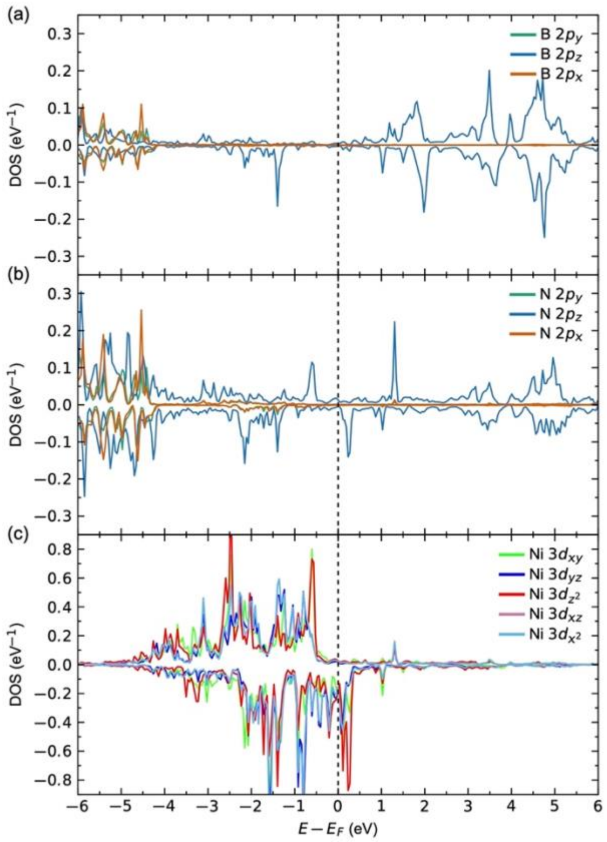

Figure S8. Orbital resolved LDOS for 1ML of pristine h-BN on Ni(111). (a) B 2*p* states, (c) N 2*p* states, and (c) Ni 3*d* states.

Supplementary Note 5: Structure perturbations from point defects

The optimized structures for intrinsic, oxygen, and carbon point defects for the three different systems are shown in Figure S9 to Figure S14. Note that the top view for all the point defect systems are directly comparable. The protruding defects are only observed for the h-BN/Ni(111) system, see Figure S10, Figure S12, and Figure S14.

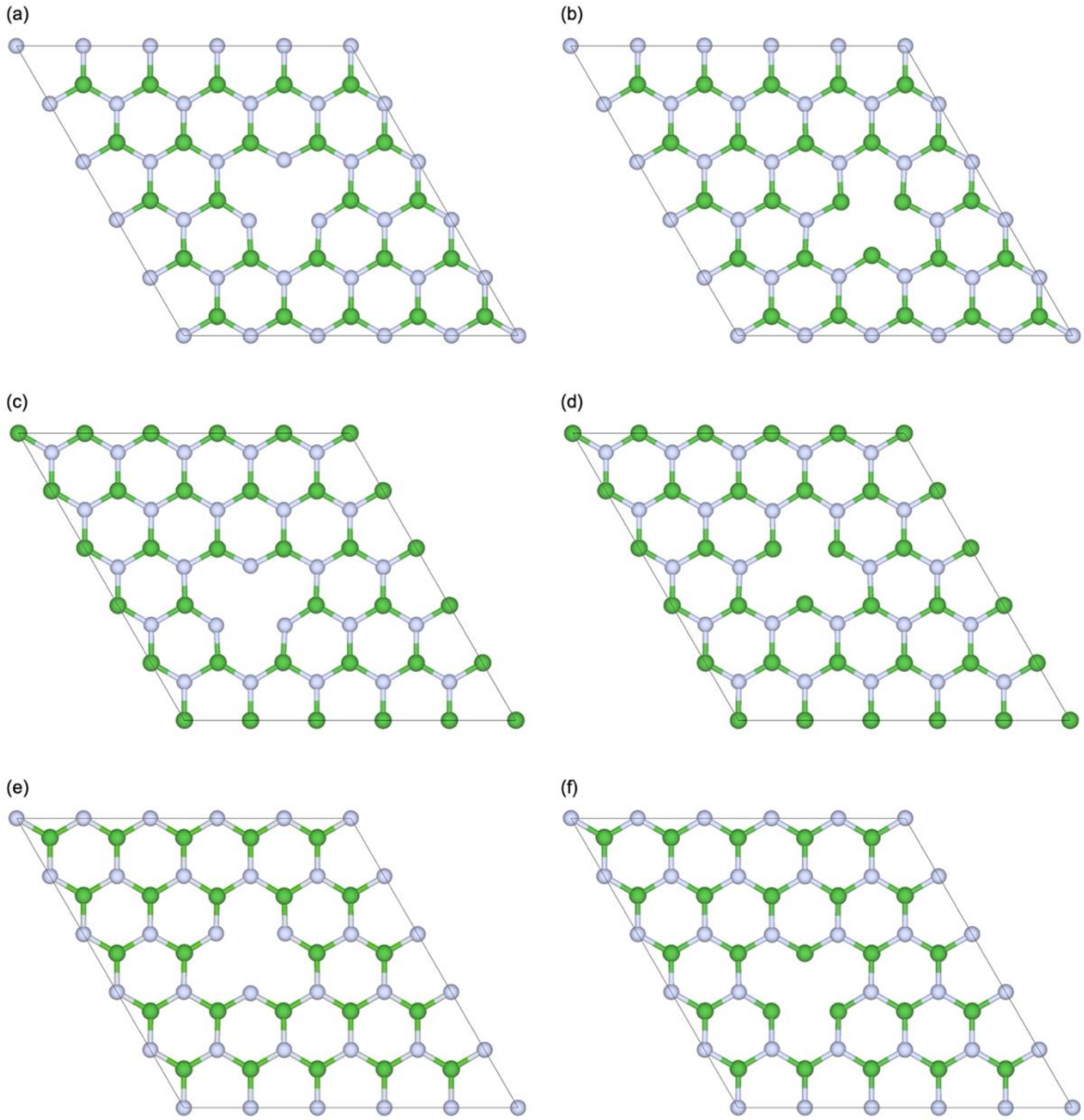

Figure S9. Top-view of the local crystal structure for (a,c,e) v_B and (b,d,f) v_N for (a,b) free-standing monolayer, (c,d) 1ML og h-BN on HOPG, and (d,e) 1ML of h-BN on Ni(111). Note that the substrate atoms in (c-f) have been omitted, for clarity.

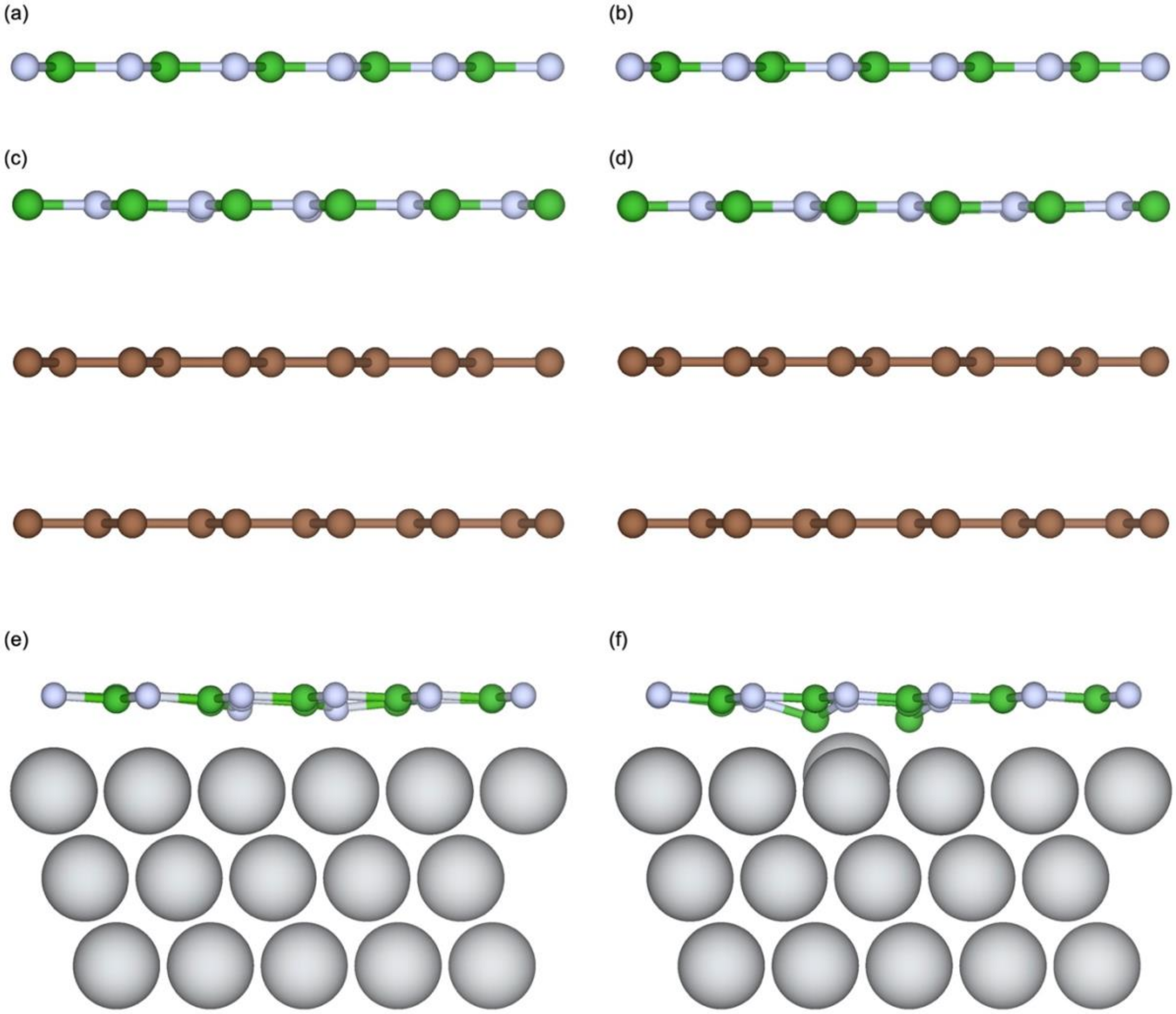

Figure S10. Side-view of the local crystal structure for (a,c,e) v_B and (b,d,f) v_N for (a,b) free-standing monolayer, (c,d) 1ML og h-BN on HOPG, and (d,e) 1ML of h-BN on Ni(111).

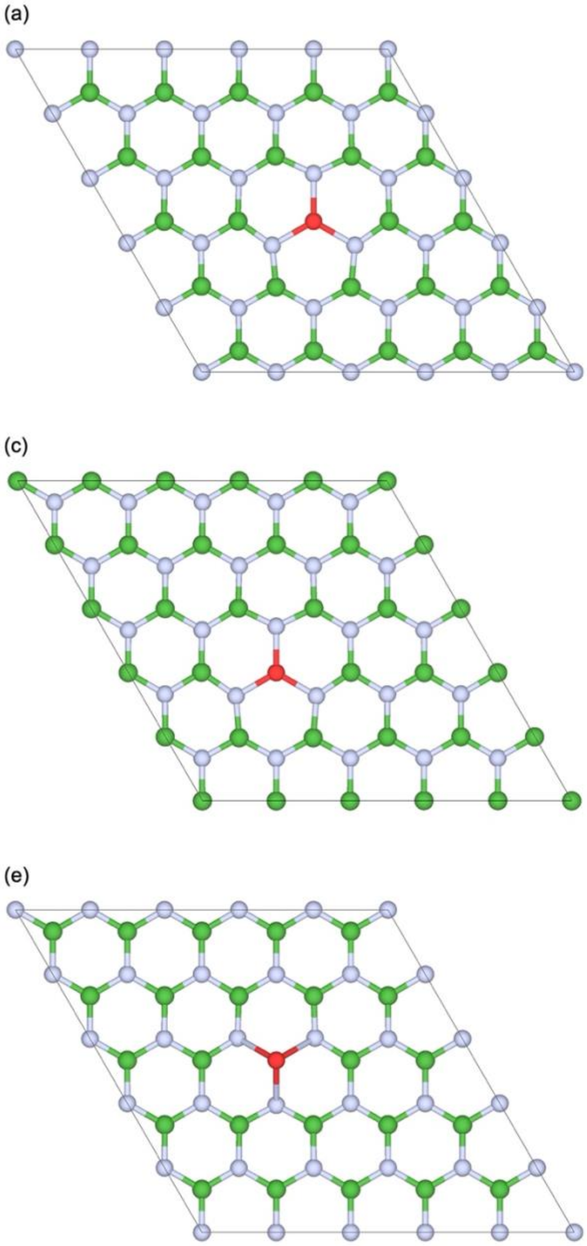

Figure S11. Top-view of the local crystal structure for (a,c,e) O_B and (b,d,f) O_N for (a,b) free-standing monolayer, (c,d) 1ML og h-BN on HOPG, and (d,e) 1ML of h-BN on Ni(111). Note that the substrate atoms in (c-f) have been omitted, for clarity.

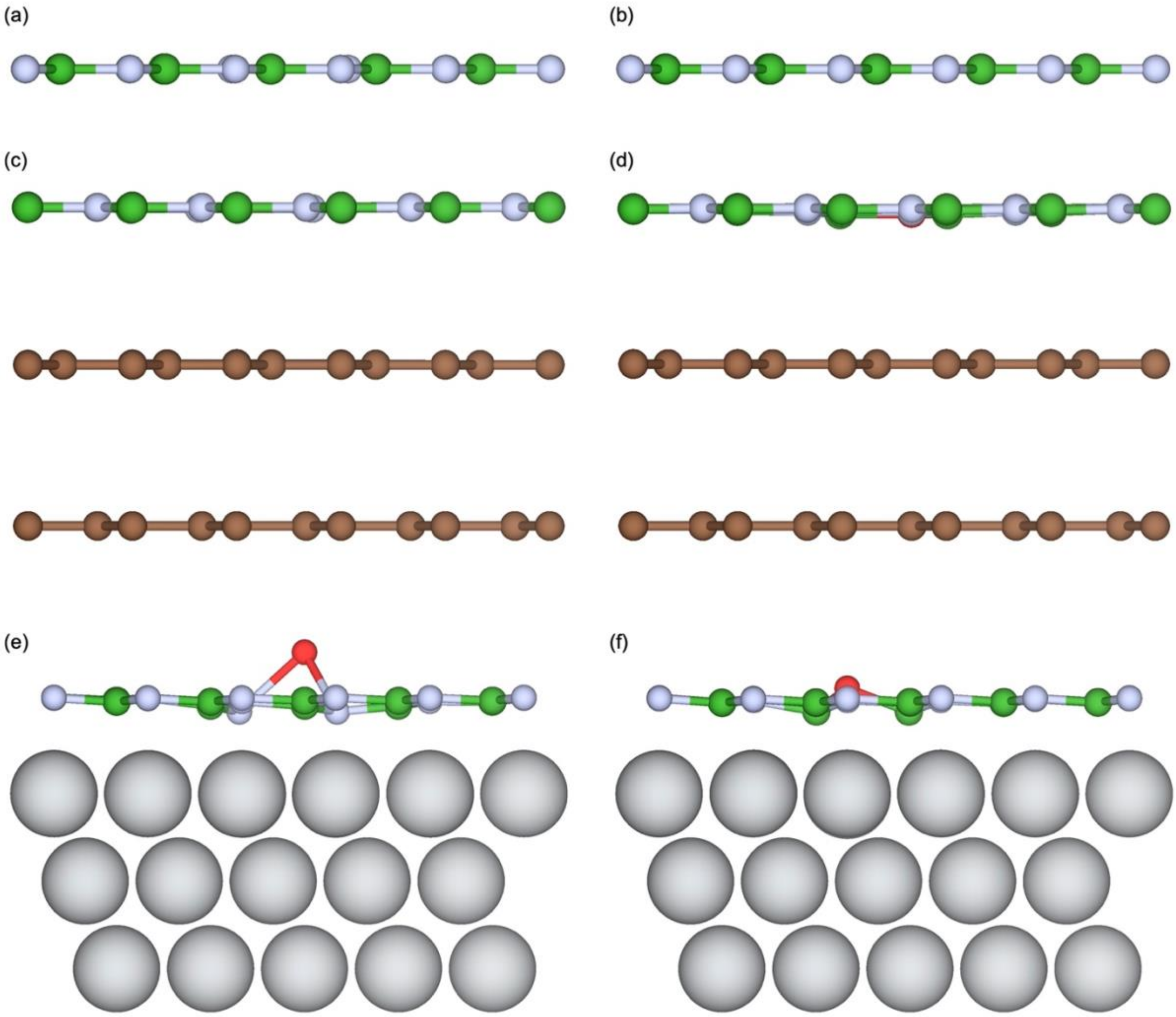

Figure S12. Side-view of the local crystal structure for (a,c,e) O_B and (b,d,f) O_N for (a,b) free-standing monolayer, (c,d) 1ML og h-BN on HOPG, and (d,e) 1ML of h-BN on Ni(111).

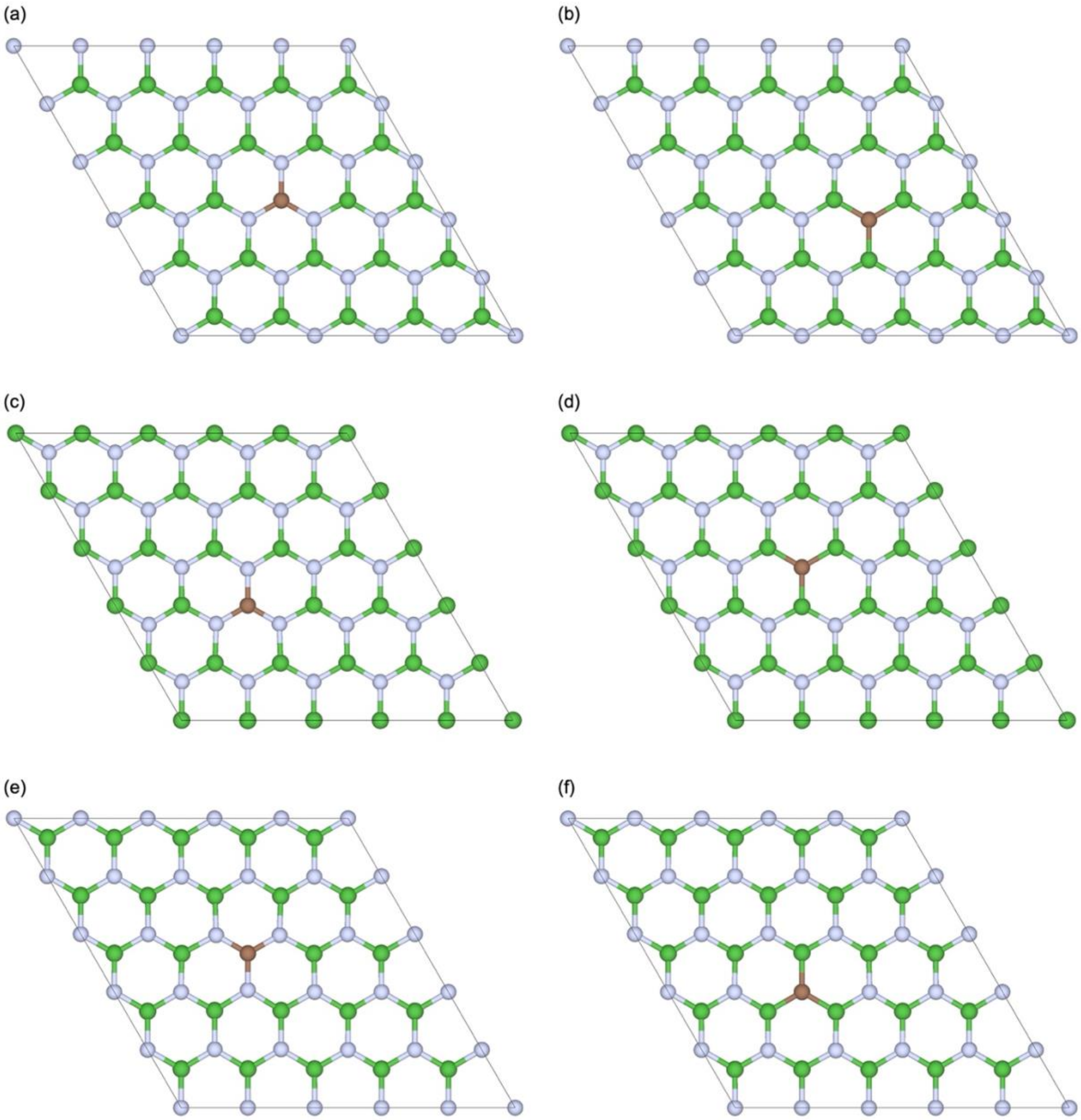

Figure S13. Top-view of the local crystal structure for (a,c,e) C_B and (b,d,f) C_N for (a,b) free-standing monolayer, (c,d) 1ML og h-BN on HOPG, and (d,e) 1ML of h-BN on Ni(111). Note that the substrate atoms in (c-f) have been omitted, for clarity.

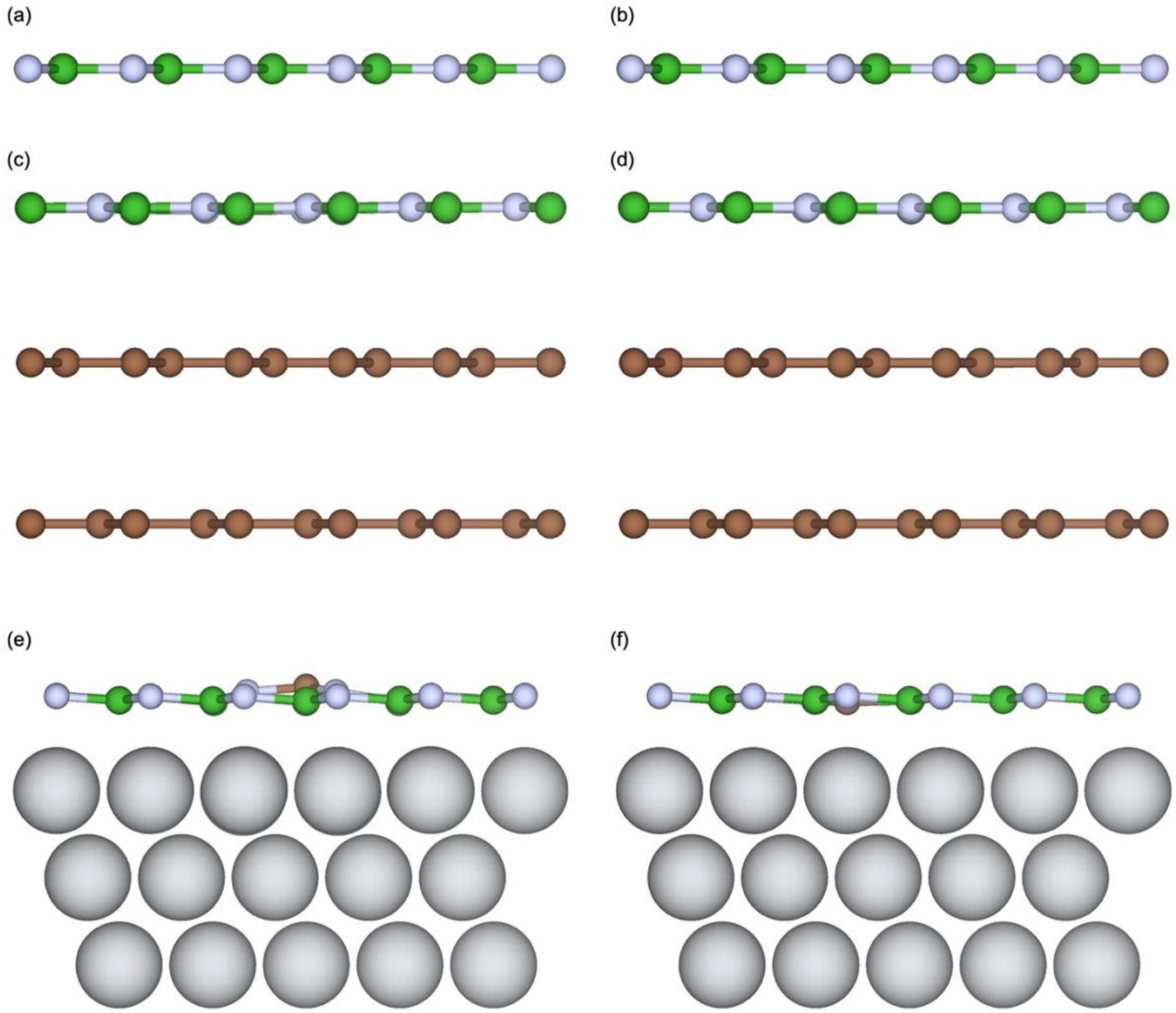

Figure S14. Side-view of the local crystal structure for (a,c,e) C_B and (b,d,f) C_N for (a,b) free-standing monolayer, (c,d) 1ML og h-BN on HOPG, and (d,e) 1ML of h-BN on Ni(111).

Supplementary Note 6. Size-dependency for in-plane h-BCN hybrid structures

To assess the graphene sheet size-dependency of the local strain associated with embedding graphene into h-BN, we have calculated the resulting crystal structure of embedding two different triangular-shaped graphene sheets *p*(8x8) supercells, illustrated in Figure S15. The configuration in Figure S15(a), labeled “C13”, consists of three six-atom carbon hexagons similar to the “C6” configuration in Figure 11(a) in the main text (marked in red). The sheet is N-terminated on the edges (marked in blue) and bonded to B at the corners (marked in black). The configuration in Figure S15(b) (“C16”) is a N-terminated only triangular shape, similar to that the “C9” configuration in Figure 11(b) in the main text, consisting of three six-atom carbon hexagons (red), edge-terminated N-C bonds (blue), and corner-terminated N-C bonds (purple). The resulting bond lengths for the two configurations are summarized in Table S9, where the B-C bonding results in large local tensile stresses in agreement with Table 7 in the main text. Figure S16 shows a comparison of calculated DOS for (a) the “C9” and (b) “C16” configurations. They show comparable electronic structures, in line with their similar structural properties.

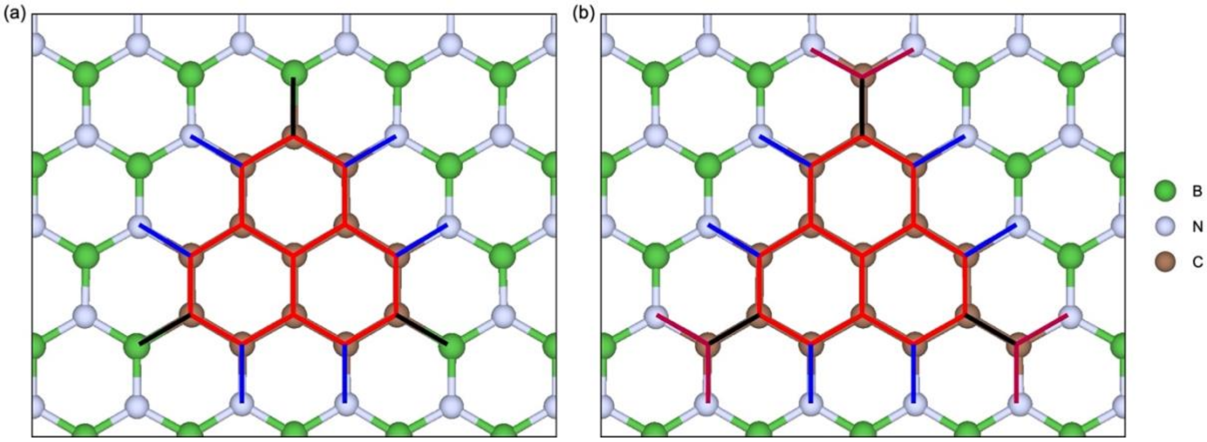

Figure S15. Top view of the two $p(8x8)$ h-BCN supercells investigated; (a) triangular shaped graphene sheet embedded in h-BN ("C13") with B-C bonds, and (b) nitrogen terminated triangular shaped graphene sheet embedded in h-BN ("C16"). Red solid lines illustrate the shape of the six-atom carbon hexagons ($(C\text{-}C)_{ring}$), blue lines N-C bonds at the edges ($(N\text{-}C)_{edge}$), black lines B-C or C-C bonds at the edges ($(B\text{-}C)_{edge}$ or $(C\text{-}C)_{edge}$), and purple N-C bonds at the corners ($(N\text{-}C)_{corner}$).

Table S9. Calculated bond lengths for the two different free-standing in-plane h-BCN configurations in *p*(8x8) supercells, similar to those in Figure 11 in the main text. Here “ring”, “edge”, and “corner” refer to the chemical bonds in the (constituting) carbon ring, at the edge of the ring, and at the corners of the triangular shape illustrated in Figure S15. Bulk B-N bulk lengths are also shown, for comparison.

	Bond length (Å)
C13	
$(C\text{-}C)_{ring}$	1.42
$(N\text{-}C)_{edge}$	1.42
$(B\text{-}C)_{edge}$	1.52
C16	
$(C\text{-}C)_{ring}$	1.43
$(C\text{-}C)_{edge}$	1.41
$(N\text{-}C)_{edge}$	1.42
$(N\text{-}C)_{corner}$	1.41
Bulk	
$(B\text{-}N)_{bulk}$	1.45

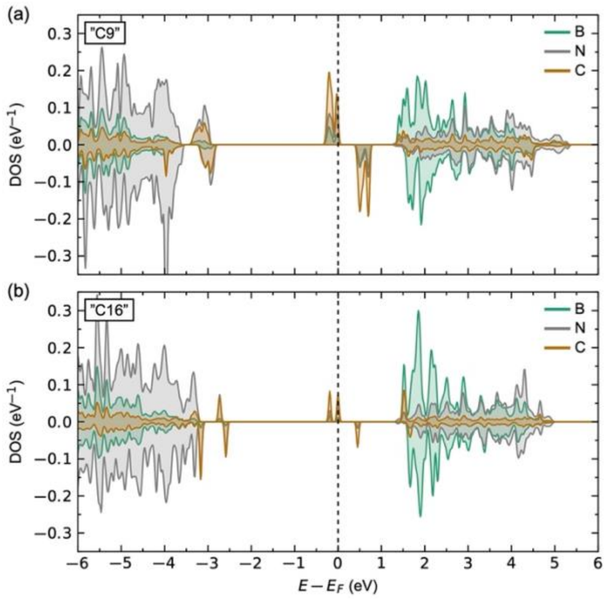

Figure S16. Calculated DOS for the (a) "C9" and (b) "C16" free-standing in-plane h-BCN hybrid configurations.

Supplementary Note 7. Stacked out-of-plane h-BN/graphene hybrid structures

Different out-of-plane h-BN/graphene stackings have been investigated, assuming 2ML thick films. Since the HOPG substrate is assumed to be bulk graphite, the only *p*(1x1) out-of-plane stacking sequence that differs from 2ML of h-BN on HOPG shown in Figure S17(a), is graphene/h-BN/HOPG (top/middle/bottom) shown in Figure S17 (b). We identify two *p*(1x1) 2ML out-of-plane stacking sequences on top of Ni(111), namely h-BN/graphene/Ni(111), graphene/h-BN/Ni(111), shown in Figure S18(b) and (c), respectively.

The interlayer distances for the different stacking sequences on HOPG and Ni(111) are summarized in Table S10, where we have also included the binding heights for 2ML of h-BN on HOPG (Figure S17(a)) and 2ML of h-BN or graphene on Ni(111) (Figure S18(a)and (d), respectively), for comparison. The relative differences in the binding heights for the different stacking sequences are in the order of 0.5 Å, and these subtle changes indicate that out-of-plane h-BN/graphene stacking should not give significant changes in the electronic properties. This becomes apparent from the corresponding LDOS for the HOPG and Ni(111) structures in Figure S19 and Figure S20, respectively, where the top-layers show comparable DOS to that of the free-standing monolayer counterparts for all systems. Some qualitative discrepancies are however observed, especially for the graphene/h-BN/Ni(111) heterostructure, which we attribute to the strong out-of-plane orbital interaction between the first layer and the substrate, and the weak out-of-plane orbital interaction between the first and second layer, as described above.

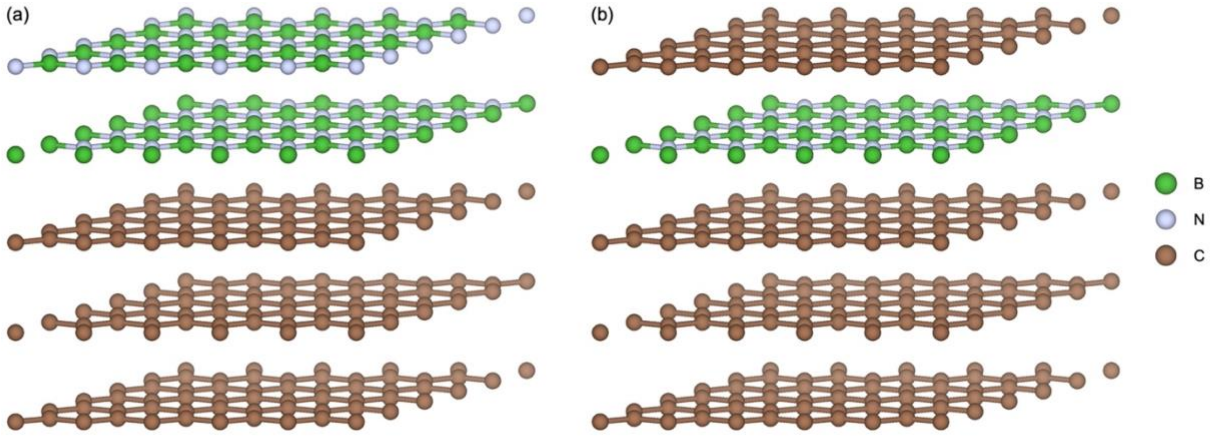

Figure S17. Calculated out-of-plane h-BN/graphene superstructures on HOPG; (a) h-BN/h-BN/HOPG, and (b) graphene/h-BN/HOPG. The three bottom layers are from the modelled HOPG substrate.

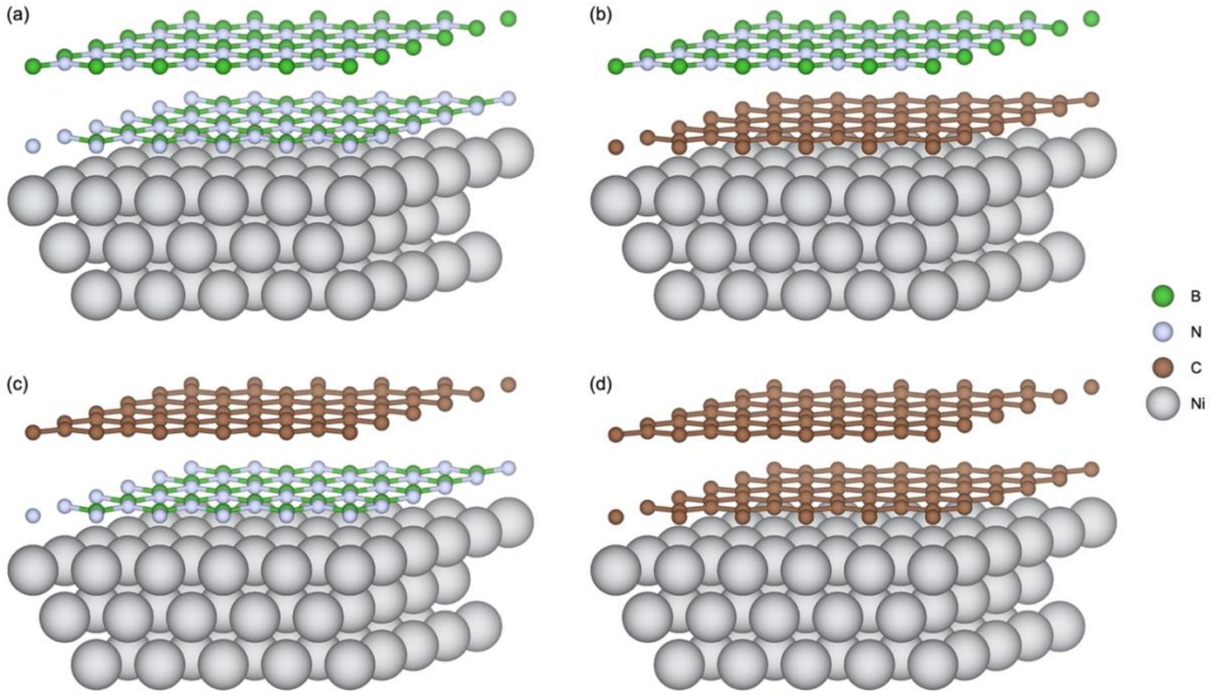

Figure S18. Calculated out-of-plane h-BN/graphene superstructures on Ni(111); (a) h-BN/h-BN/Ni(111), (b) h-BN/graphene/Ni(111), (c) graphene/h-BN/Ni(111), and (d) graphene/graphene/Ni(111). The three bottom layers are from the modelled Ni(111) substrate.

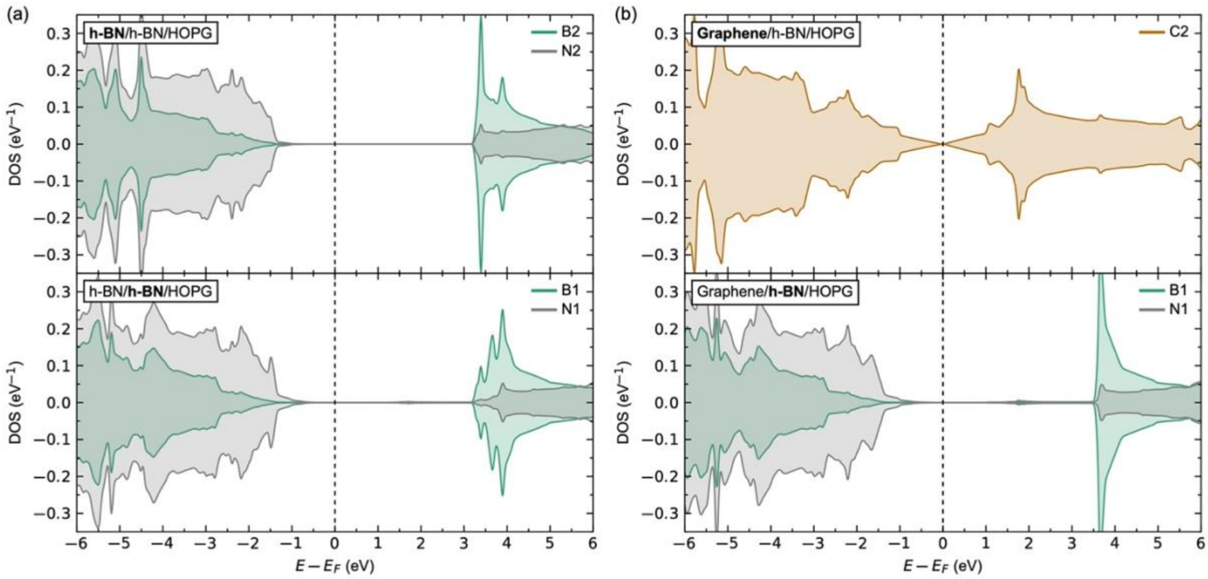

Figure S19. Calculated LDOS for the different out-of-plane h-BN/graphene superstructures on HOPG; (a) h-BN/h-BN/HOPG, and (b) graphene/h-BN/HOPG. The numbers indicate the first or second monolayer of h-BN or graphene starting from the closest one to the substrate interface. The bold text refers to the respective atomic layer.

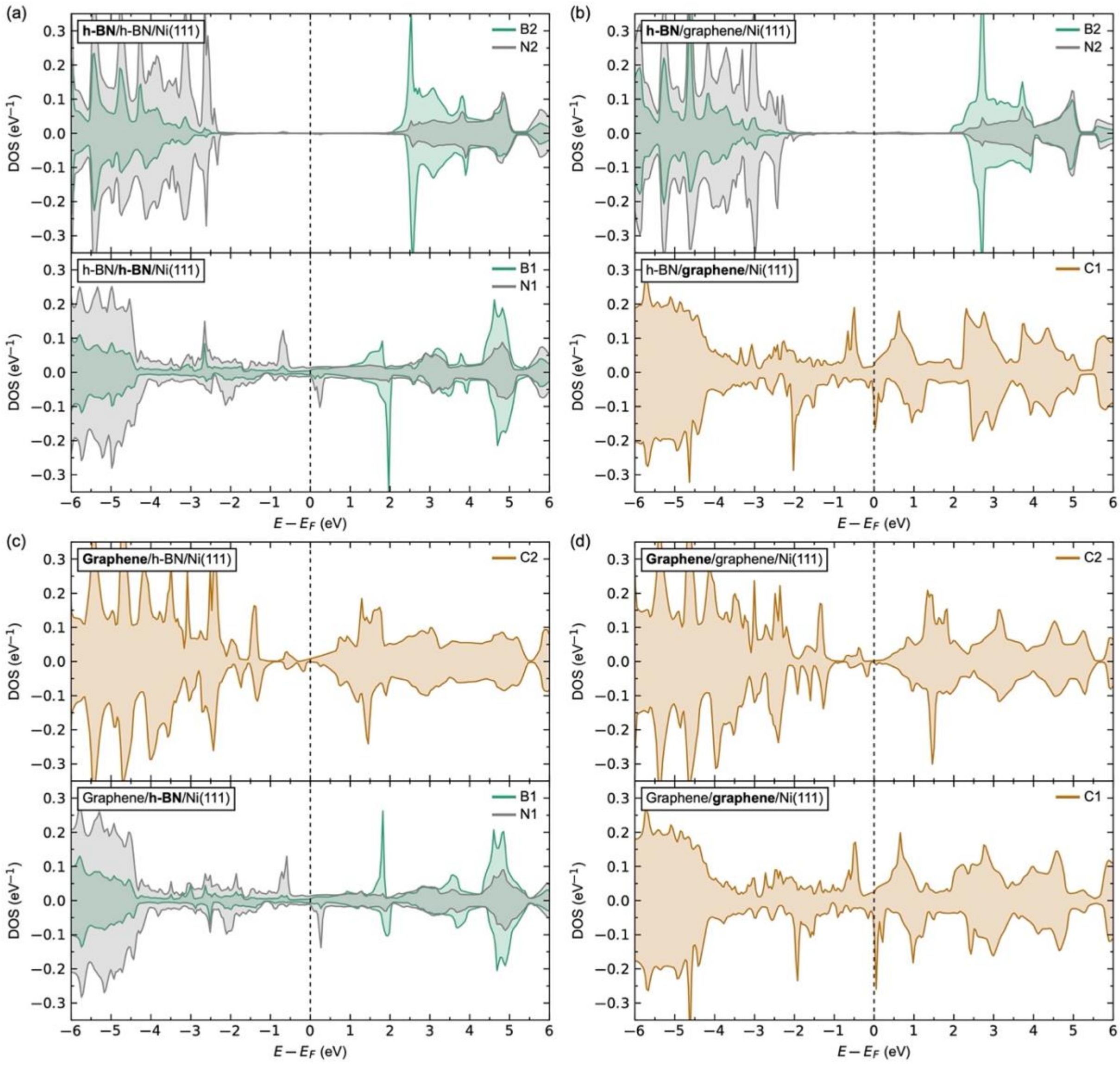

Figure S20. Calculated LDOS for the different out-of-plane h-BN/graphene superstructures on Ni(111); (a) h-BN/h-BN/Ni(111), (b) h-BN/graphene/Ni(111), (c) graphene/h-BN/Ni(111), and (d) graphene/graphene/Ni(111). The numbers indicate the first or second monolayer of h-BN or graphene starting from the closest one to the substrate interface. The bold text refers to the respective atomic layer.

Table S10. Calculated interlayer distances for different out-of-plane h-BN/graphene stacking sequences on HOPG and on Ni(111). $h_{0,1}$ corresponds to the distance between the substrate and the first monolayer, and $h_{1,2}$ corresponds to the distance between the first and second monolayers from the substrate.

Layer	Interlayer distance (Å)					
	HOPG		Ni(111)			
	h-BN/ h-BN/	Graphene/ h-BN/	h-BN/ h-BN/	h-BN/ Graphene /	Graphene / h-BN/	Graphene / Graphene /
$h_{0,1}$	3.26	3.25	2.12	2.13	2.13	2.12
$h_{1,2}$	3.27	3.26	3.22	3.20	3.22	3.27

References

[1] M. Dion, H. Rydberg, E. Schröder, D.C. Langreth, and B.I. Lundqvist, "Van der Waals Density Functional for General Geometries," Phys. Rev. Lett. **92**(24), 246401 (2004).

[2] G. Román-Pérez, and J.M. Soler, "Efficient implementation of a van der waals density functional: Application to double-wall carbon nanotubes," Phys. Rev. Lett. **103**(9), 096102 (2009).

[3] K. Lee, É.D. Murray, L. Kong, B.I. Lundqvist, and D.C. Langreth, "Higher-accuracy van der Waals density functional," Phys. Rev. B - Condens. Matter Mater. Phys. **82**(8), 081101 (2010).

[4] J. Klimeš, D.R. Bowler, and A. Michaelides, "Chemical accuracy for the van der Waals density functional," J. Phys. Condens. Matter **22**(2), 022201 (2010).

[5] I. Hamada, "Van der Waals density functional made accurate," Phys. Rev. B - Condens. Matter Mater. Phys. **89**(12), 121103 (2014).

[6] H. Peng, Z.H. Yang, J.P. Perdew, and J. Sun, "Versatile van der Waals density functional based on a meta-generalized gradient approximation," Phys. Rev. X **6**(4), 041005 (2016).

[7] J.P. Perdew, A. Ruzsinszky, G.I. Csonka, O.A. Vydrov, G.E. Scuseria, L.A. Constantin, X. Zhou, and K. Burke, "Restoring the Density-Gradient Expansion for Exchange in Solids and Surfaces," Phys. Rev. Lett. **100**(13), 136406 (2008).

[8] J.P. Perdew, K. Burke, and M. Ernzerhof, "Generalized Gradient Approximation Made Simple," Phys. Rev. Lett. **77**(18), 3865–3868 (1996).

[9] J.P. Perdew, and A. Zunger, "Self-interaction correction to density-functional approximations for many-electron systems," Phys. Rev. B **23**(10), 5048–5079 (1981).

[10] L. Liu, Y.P. Feng, and Z.X. Shen, "Structural and electronic properties of h -BN," Phys. Rev. B **68**(10), 104102 (2003).

[11] C. Tarrio, and S.E. Schnatterly, "Interband transitions, plasmons, and dispersion in hexagonal boron nitride," Phys. Rev. B **40**(11), 7852–7859 (1989).

[12] K. Watanabe, T. Taniguchi, and H. Kanda, "Direct-bandgap properties and evidence for ultraviolet lasing of hexagonal boron nitride single crystal," Nat. Mater. **3**(6), 404–409 (2004).

[13] P. Trucano, and R. Chen, "Structure of graphite by neutron diffraction," Nature **258**(5531), 136–137 (1975).

[14] Z. Wang, S.M. Selbach, and T. Grande, “Van der Waals density functional study of the energetics of alkali metal intercalation in graphite,” RSC Adv. **4**(8), 4069–4079 (2014).

[15] W.P. Davey, “Precision measurements of the lattice constants of twelve common metals,” Phys. Rev. **25**(6), 753–761 (1925).

[16] G. Giovannetti, P.A. Khomyakov, G. Brocks, P.J. Kelly, and J. Van Den Brink, “Substrate-induced band gap in graphene on hexagonal boron nitride: Ab initio density functional calculations,” Phys. Rev. B - Condens. Matter Mater. Phys. **76**(7), 073103 (2007).

[17] B. Grad, P. Blaha, K. Schwarz, W. Auwärter, and T. Greber, “Density functional theory investigation of the geometric and spintronic structure of h-BN/Ni(111) in view of photoemission and STM experiments,” Phys. Rev. B - Condens. Matter Mater. Phys. **68**(8), 085404 (2003).

[18] R. Laskowski, P. Blaha, and K. Schwarz, “Bonding of hexagonal BN to transition metal surfaces: An ab initio density-functional theory study,” Phys. Rev. B - Condens. Matter Mater. Phys. **78**(4), 045409 (2008).

[19] A.A. Tonkikh, E.N. Voloshina, P. Werner, H. Blumtritt, B. Senkovskiy, G. Güntherodt, S.S.P. Parkin, and Y.S. Dedkov, “Structural and electronic properties of epitaxial multilayer h-BN on Ni(111) for spintronics applications,” Sci. Rep. **6**(1), 23547 (2016).

[20] M.N. Huda, and L. Kleinman, “h - BN monolayer adsorption on the Ni(111) surface: A density functional study,” Phys. Rev. B **74**(7), 075418 (2006).

[21] X. Sun, A. Pratt, Z.Y. Li, M. Ohtomo, S. Sakai, and Y. Yamauchi, “The adsorption of h -BN monolayer on the Ni(111) surface studied by density functional theory calculations with a semiempirical long-range dispersion correction,” J. Appl. Phys. **115**(17), 17C117 (2014).

[22] G. Kim, S.C. Jung, and Y.K. Han, “Selectively strong molecular adsorption on boron nitride monolayer induced by transition metal substrate,” Curr. Appl. Phys. **13**(9), 2059–2063 (2013).

[23] A. Lyalin, A. Nakayama, K. Uosaki, and T. Taketsugu, “Functionalization of monolayer h-BN by a metal support for the oxygen reduction reaction,” J. Phys. Chem. C **117**(41), 21359–21370 (2013).

[24] A.H.M.A. Wasey, S. Chakrabarty, G.P. Das, and C. Majumder, “*h* -BN Monolayer on the Ni(111) Surface: A Potential Catalyst for Oxidation,” ACS Appl. Mater. Interfaces **5**(21), 10404–10408 (2013).

[25] R. Koitz, J.K. Nørskov, and F. Studt, "A systematic study of metal-supported boron nitride materials for the oxygen reduction reaction," Phys. Chem. Chem. Phys. **17**(19), 12722–12727 (2015).

[26] Y. Guo, and W. Guo, "Hydroxylation of a metal-supported hexagonal boron nitride monolayer by oxygen induced water dissociation," Phys. Chem. Chem. Phys. **17**(25), 16428–16433 (2015).
